# Automated stopped-flow library synthesis for rapid optimisation and machine learning directed experimentation†

**DOI:** 10.1039/d2sc03016k

**Published:** 2022-09-13

**Authors:** Claudio Avila, Carlo Cassani, Thierry Kogej, Javier Mazuela, Sunil Sarda, Adam D. Clayton, Michael Kossenjans, Clive P. Green, Richard A. Bourne

**Affiliations:** Sample Management, Discovery Sciences, BioPharmaceuticals R&D, AstraZeneca Cambridge CB4 0WG UK Claudio.avila@science.cl R.A.Bourne@leeds.ac.uk; Research and Early Development, Medicinal Chemistry Respiratory & Immunology Göteborg Sweden; MolecularAI, Discovery Sciences, R&D, AstraZeneca Göteborg Sweden; Institute of Process Research and Development, School of Chemistry and School of Chemical and Process Engineering, University of Leeds Leeds LS2 9JT UK

## Abstract

For the discovery of new candidate molecules in the pharmaceutical industry, library synthesis is a critical step, in which library size, diversity, and time to synthesise are fundamental. In this work we propose stopped-flow synthesis as an intermediate alternative to traditional batch and flow chemistry approaches, suited for small molecule pharmaceutical discovery. This method exploits the advantages of both techniques enabling automated experimentation with access to high pressures and temperatures; flexibility of reaction times, with minimal use of reagents (μmol scale per reaction). In this study, we integrate a stopped-flow reactor into a high-throughput continuous platform designed for the synthesis of combinatory libraries with at-line reaction analysis. This approach allowed ∼900 reactions to be conducted in an accelerated timeframe (192 hours). The stopped flow approach used ∼10% of the reactants and solvents compared to a fully continuous approach. This methodology demonstrates a significantly improved synthesis success rate of smaller libraries by simplifying the implementation of cross-reaction optimisation strategies. The experimental datasets were used to train a feed-forward neural network (FFNN) model providing a framework to guide further experiments, which showed good model predictability and success when tested against an external set with fewer experiments. As a result, this work demonstrates that combining experimental automation with machine learning strategies can deliver optimised analyses and enhanced predictions, enabling more efficient drug discovery investigations across the design, make, test and analysis (DMTA) cycle.

## Abstract 2

Combining experimental stopped flow automation with machine learning strategies can deliver optimised conditions and enhanced predictions, enabling more efficient design, make, test and analysis (DMTA) cycles.

## Graphical abstract

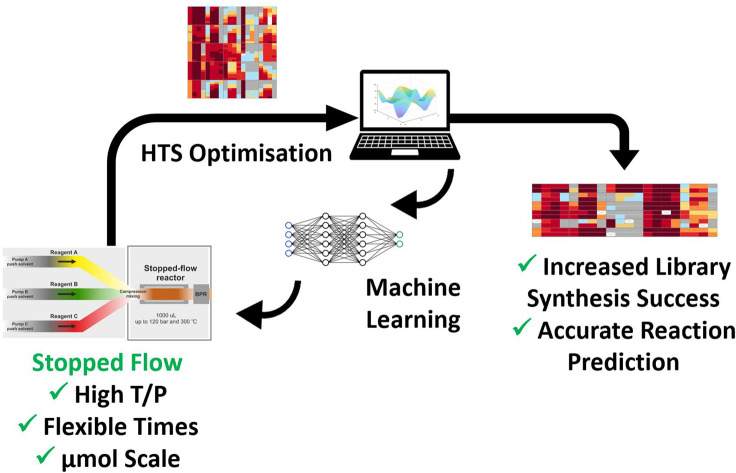

## 1 Introduction

Diversity-oriented synthesis (DOS) is one of the main strategies used in early drug discovery to generate libraries of molecules, targeting the identification of biologically active compounds.^1,2^ The creation of a library follows an iterative cycle of compound design, make, test, and analysis (DMTA cycle), directed from the information obtained from bioassays and structure–activity relationship (SAR) analysis back to the compound design stage. This iterative process is used to generate a set of molecules with improved biological activity with the potential for clinical development.^3^ Several parameters for building the ideal library have been identified including library diversity, from which scaffold diversity is more relevant than the peripheral substituents,^4^ and the library size. Many approaches to synthesise combinatory libraries have been tested,^5–7^ although few have considered their integration into the iterative DMTA cycle enabling the study and optimisation of the key characteristic features of the ideal library.

For a single DMTA iteration, the overall efficiency of the synthesis step can be linked to several factors. From an experimental perspective, the work to synthesise a library starts with the combination of arrays of reagents to generate a set of backbone structures^8^*e.g.* for an amide coupling derived library, combining a set of carboxylic acids with a set of amines.^9^ At this stage the number of reactions is large, often performed in parallel and under the same reaction conditions.^10^ In this context, traditional batch methods (limited to relatively large volumes and reaction times) compete with modern high-throughput approaches such as parallel scaffold synthesis on well plates (limited to mild reaction conditions and compatible solvents),^11,12^ both leaving large areas of the parameter space unexplored. In these systems, the average library success rate varies from 50% to 80%, representing a first outcome that can be improved.^13^

Another feature that can lead to a higher success rate is tailoring synthetic conditions based on the information available.^14^ Synthetic knowledge is generated during each DMTA cycle or from literature precedent, including past reaction conditions, the suitability of using specific reagents and solvents, and individual steps required for specific transformations (methods).^15,16^ In addition, the analytical characterisation of the reaction results provides information about the reaction success including selectivity, purity, and yields.^17^ Traditionally, this information is gathered by the synthetic chemists as ‘know-how’ or written in laboratory notebooks, and just recently the use of machine learning approaches has allowed to create models to exploit this to a greater extent.^18^

Emerging machine learning strategies make use of large experimental datasets such as those found in proprietary Electronic Laboratory Notebooks (ELN), the USPTO database, Reaxys, *etc.*^19^ However, the same datasets applied to the prediction of suitable reaction conditions have reached limited success.^20^ This is partly due to the absence of standardisation of recording input parameters such as reaction times, reaction volumes, concentrations, and accuracy to record the synthesis conditions, making these models to diverge considerably from reality when the reactions are unknown, or when the reaction substrates are not present in the training sets. This is further compounded by the generally poor reproducibility of experimental work held within such datasets. As a result, the experimental synthesis of a drug discovery library driven by a pure algorithmic use of the information (data capture, analyse, learn, apply) has not been materialised yet.

Recent advances in automation and flow chemistry have brought this concept closer.^21–24^ In this, chemical reactions are performed under continuous flow, providing safe access to novel process windows (high temperatures and pressures, above solvents boiling point),^25^ maximising mass and heat transfer per unit of volume, highly precise control and ease of automation.^26,27^ These advantages have been exploited in several flow platforms developed for synthesis and manufacturing, involving process engineering and reaction optimisation,^5,28^ which were also applied to synthesise compound libraries.^29^ Moreover, this approach facilitates the integration of automation algorithms for searching the chemical space systematically.^30^ Despite these benefits, continuous flow chemistry still shows some disadvantages for medicinal chemistry such as the synthesis scale (relatively high), requiring to pump large reagent volumes to achieve steady state conditions,^31^ with unnecessary environmental and economic costs *e.g.* when using hazardous or expensive reagents.^32^ For small volumes (microreactors), the use of dedicated and expensive equipment is necessary to achieve low flow rates required for relatively long residence times.^33^

Stopped-flow reactors provide an alternative method for library synthesis. In this approach, the slugs of substrates are premixed and channelled to the reactor (same as in continuous flow), stopping the flow when the reagents reach the centre of the reactor, and waiting for a specific reaction time to elapse. The methodology was developed in the field of analytical chemistry for kinetic determinations (1940s), adapting from the recently established continuous flow methods to achieve a more economic use of reagents.^34^ The applications range from small molecule kinetics up to determination of enzyme kinetics,^35^ and some advantages include:

• Provides an experimental framework that can be standardised to replicate experimental conditions independently from the available hardware.

• For a stopped-flow reactor the accessible residence time is independent of the flow rate, hence any pump that operates within the desired pressure range can be used (see ESI†).

• It provides access to a wider array of pressures and temperatures, inaccessible for batch, well plates, and traditional flow methods.

• A small reactor/slug size directly relates to a lower use of reagents and solvents, with a much lower waste generation compared to continuous flow and batch methods.

• It avoids sample cross-contamination, and the use of gas or perfluorinated solvents.

• Process intensification (*via* temperatures above the solvent boiling point or temperatures where the second phase dissolve) and reaction throughput can be increased substantially enhanced compared to biphasic segmented flow.^36^

• It has similar benefits for mass and energy transfer compared with continuous flow.

• Small working volumes enable easier downstream separation.

In the same way as used in traditional flow chemistry, stopped-flow rectors can be integrated with a liquid handler unit and used for high-throughput library synthesis. Moreover, in this work we demonstrate their use, integration with self-optimisation algorithms, and the extraction of characteristic reaction outcome features that can be used to optimise the complete library synthesis step.

## 2 Results and discussion

### 2.1 Platform design

**Fig. 1:**
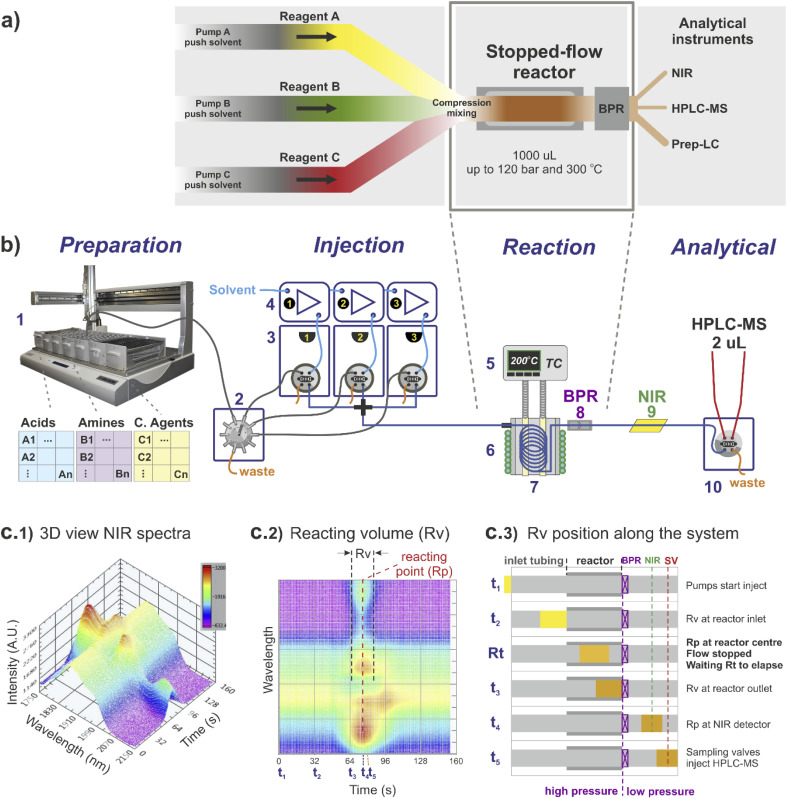
Stopped-flow reactor integrated into a continuous high-throughput platform: (a) stopped-flow system concept. The reactor is built by a small diameter coil, with the substrates injected simultaneously. The flow is stopped when material reaches the reaction point, and output for analysis/recovery when the desired ‘reaction time’ has elapsed; (b) high-throughput platform: (1) liquid handler; (2) multi-selection valve; (3) array of sampling loops, each connected to a (4) respective HPLC pump; (5) reactor temperature digitally controlled; (6) cooling jacket digitally controlled; (7) stopped-flow reactor coil; (8) back pressure regulator; (9) NIR flow cell; (10) 2 μL sampling loop connected to HPLC-MS. (c) Calibration and control of the stopped-flow reactor using NIR signal: (c1) 3D NIR signal of a single reaction; (c2) 2D NIR signal showing relative location of Rv (reaction volume) and Rp (reacting point); (c3) time position of the Rv along the system from injection (*t*_1_) to reaction (Rt) and sampling (*t*_5_).

The stopped-flow reactor configuration provides similar mass and energy transfer conditions compared to continuous flow at the scales required for library synthesis (Fig. 1). One factor that differs from the continuous flow is the concept of residence time. For the stopped-flow reactor, ‘reaction time’ (Rt) is a more appropriate term (analogous to a batch system), formed by the contribution of residence time whilst flowing *i.e.*, calculated using the flow rate to move the material into and out of the reactor (same as in continuous flow), added to the time in which the reacting volume is kept motionless. As a result, the reaction time is flexible, independent of the flow rate capacity provided by the pumping instruments and much less reliant on the operational performance of the specific pump used compared to traditional flow systems.

For reference, the centre of the reacting volume position is referred as the ‘reacting point’ (Rp), monitored along the length of the flow system using a low cost NIR sensor (Fig. 1c), effectively differentiating between the ‘reaction crude’ (substrates dissolved in solvent A) and the pushing/flushing solvent (solvent B) used to move the reaction crude in-and-out of the reactor (Fig. 1c2 and c3).

Regarding the use of solvents and reagents, a ∼90% reduction was achieved using a stopped-flow reactor compared to a fully continuous system. For instance, to produce 0.5 mL in a 1 mL reactor (synthesis scale of screening library), the stopped-flow reactor required ∼1 mL of reacting volume (Rv), assuming twice the required volume to eliminate any potential axial interference, whilst in continuous flow the same quantity would require a total of ∼10 mL of reagents (reaching steady-state conditions before sampling).

### 2.2 Searching for optimal reaction conditions

The stopped-flow reactor was integrated into a continuous platform that enabled the systematic exploration of variables which could be relevant for the reactions. For the libraries synthesised at the initial iterations of the DMTA cycle, the successful generation of candidate molecules is essential. At these early stages, libraries are large and focussed on building diversity, from which each successful molecule would be the base to create further analogues.

**Fig. 2:**
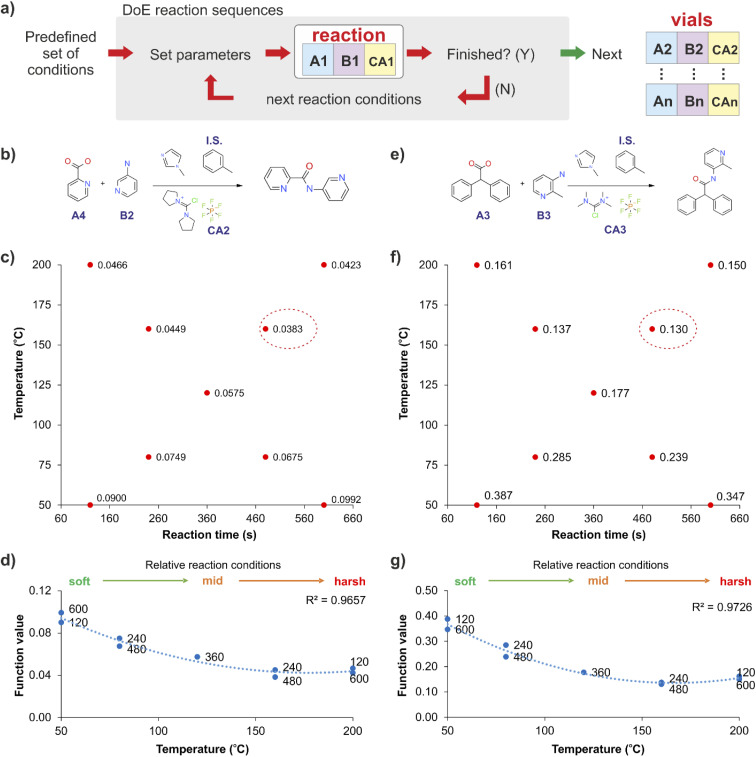
Simplified exploration of the reaction conditions (a) using a fast design of experiments (DoE) pattern applied to two reactions (b and e). The fast DoE explores nine reaction conditions (c and f), indicating the minimisation function value (the ratio of the internal standard to the product peak areas, *i.e.* with increasing product area this will reduce, calculated from the HPLC DAD 254 nm signal) by a red circle. (d) and (g) show the strong temperature dependency for the optimum reaction conditions (when the function value is minimum), with a negligible influence of the reaction time.

For these stages we propose the use of a predefined set of physical conditions (a fast design of experiments (DoE) approach in temperature and reaction time), to identify ‘adequate’ synthesis conditions, and releasing experimental time for screening chemical variables. This autonomous predefined DoE method was chosen over a machine learning directed optimisation approach, SNOBFIT (see ESI, section S3†), as the data collected for new combinations was comparable at identical conditions and required less experimentation (see Fig. S3†). Fig. 2 shows the results obtained by applying this DoE method to two reactions (Fig. 2b and e), producing models that are suitable to establish cross-correlations between different reactions across the whole library. The method was simplified further by reducing the bi-dimensionality of the ‘temperature-reaction time’ to a linear pattern, ranging from mild conditions (low temperature and short reaction time) to harsher conditions (high temperature and long reaction time). This classification was specific for the reactions used in this study, but the methodology can be transferred to other systems. This approach was effective in identifying suitable synthesis conditions, which in general showed a strong temperature dependency and a reduced influence of the reaction time for the optimum reaction conditions (Fig. 2d and g).

### 2.3 High-throughput library synthesis using stopped-flow

**Scheme 1:**
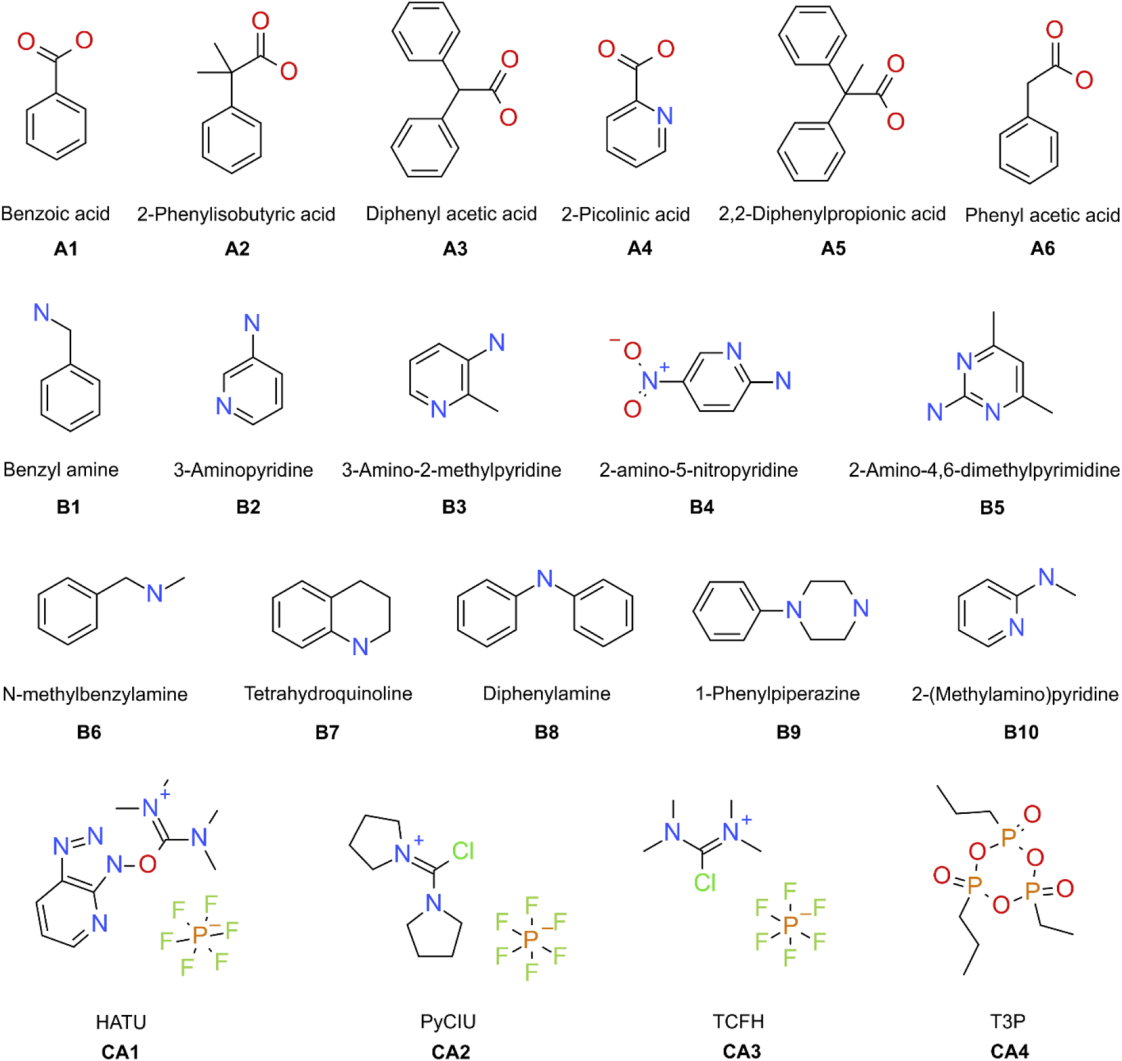
Carboxylic acids (A), amines (B), and coupling agents (CA) used in this study.

**Fig. 3:**
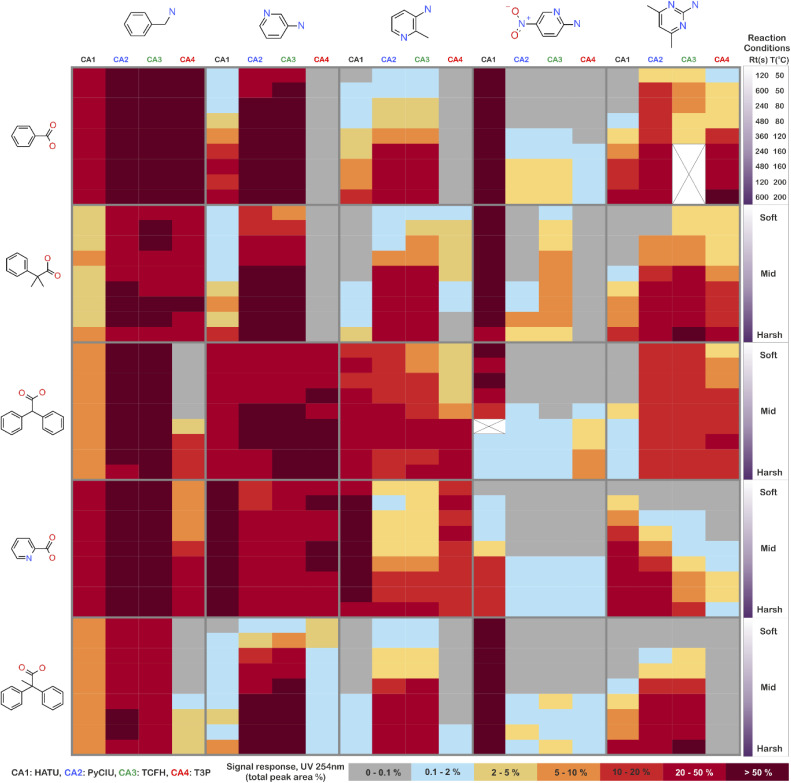
Heatmap for the amide library synthesised. Five carboxylic acids were combined with five amines, using four coupling agents and tested under nine different reaction conditions (900 individual reactions). Colours indicate the relative abundance of the target molecule (UV peak area of the target molecule signal relative to the total chromatogram area (%), measured at 254 nm). White and crossed boxes indicate absence of analytical data due to instrument failure.

The stopped-flow reactor was used in high-throughput mode (continuous platform) to synthesise an amide library of 25 components, combining five acids (Scheme 1, A1 to A5; from which 4 contained sterically hindered reaction centres) and five amines (Scheme 1, B1 to B5; from which 4 were electron deficient nucleophiles). Four different coupling agents were used (Scheme 1, CA1 to CA4) including traditional HATU and T3P, compared against the use of PyCIU (providing *in situ* formation of acyl chlorides)^37^ and TCFH-NMI (providing direct access to *N*-acyl imidazoliums),^38^ both suitable for challenging amide bond formations. Each of the 25 combinations of acid and amine were subjected to the four coupling agents and nine reaction conditions, performing a total of 900 individual reactions which were completed in ∼192 hours. Fig. 3 shows the library synthesised in the form of a heatmap, where the assay conversion to product is indicated by the UV peak area of the target molecule signal relative to the total chromatogram area (%). In Fig. 3, a grid of experiment results is shown with the major columns showing each of the 5 amines and minor columns within this being the four coupling agents. The 5 major rows show the five carboxylic acids with the 9 minor rows showing soft (120 s, 50 °C) up to harsh (600 s, 200 °C) reaction conditions, finally conversion to product is indicated by the colours in this heatmap.

The calculation of an absolute yield (obtained from the isolated target product) is typically a very much an essential parameter during reaction optimisation. However, reactions performed for library synthesis are deemed successful if they produce enough material with viable recovery to perform a bioassay. Biological testing and sample retention following synthesis typically requires a recovery of 2.5 mg of product per reaction, and this information is inferred directly from the HPLC signal assuming it always occurs when the target molecule area is larger than 8–10% of the total area. The optimal reaction conditions observed differed considerably from those previously reported in the literature. In particular, the reaction time explored was relatively short (2 to 10 min range), compared to previous experiments that deployed the same reactions in batch mode, which varied from 30 min to 24 hours.^37,38^ This difference could be partly responsible for the limited conversion achieved in some specific transformations. In contrast, the stopped-flow reactor allowed the exploration of a broader temperature range (50–200 °C), normally limited by the boiling point of the solvent used when working in traditional batch synthesis (80–140 °C) or equipment limitations for high throughput platforms (typically 20–70 °C). In these cases, the pressure of the system reached up to ∼100 bar at the highest temperature. Even at the most extreme conditions, the BPR avoided the formation of vapours and bubbles during the reaction. This was confirmed by the NIR readings at the reactor end, which is highly sensitive to the appearance of any interface that creates spectral noise.

### 2.4 Effectiveness of the coupling agent

**Fig. 4:**
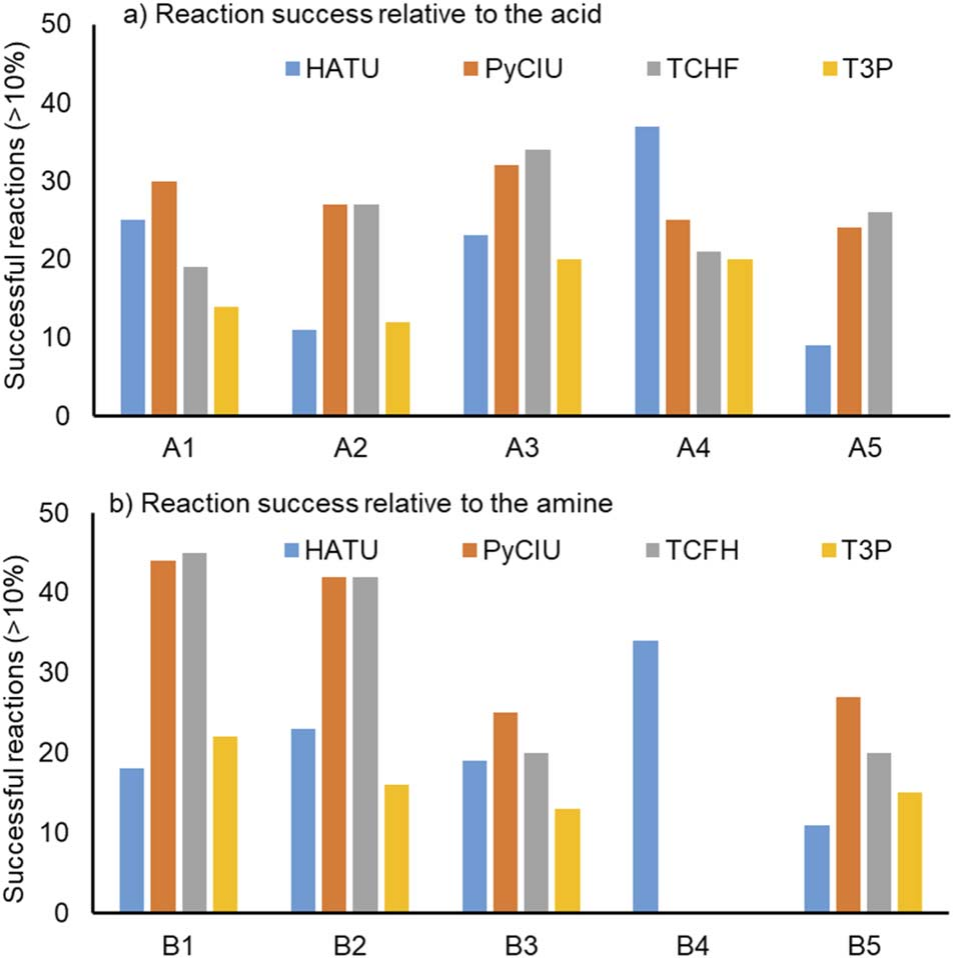
Effectiveness of the coupling agent relative to each of the substrates used, considering successful reactions those in which the desired product produced a signal >10% of the total chromatogram area (measured at UV DAD 254 nm signal).

Fig. 4 shows two graphs comparing their effectiveness relative to each of the substrates (where the targeted molecules had a UV peak area >10% of the total chromatogram), separating between acids and amines. In general, HATU and T3P were overall less successful, producing dirtier reactions (accompanied by the formation of a large quantity of side-products), particularly accentuated when the temperature was above 120 °C. These conditions were also detrimental for the chromatographic separation, which combined with the influence of the base used to activate the acid (to promote the reaction mechanism), degraded the chromatographic column during the sequences, which then required column regeneration. This affected the separation efficiency, with many impurities coeluting alongside the desired product. Conversely, PyCIU and TCFH show better performance in terms of the number of successful reactions, a lower generation of side products, and less aggressive reaction conditions. This allowed good chromatographic separation, although in some cases impurities also coeluted with the desired product. Amongst all the substrates and coupling agents used, 2-amino-5-nitropyridine (B4) was the most challenging amine. This amine was particularly difficult to react and had a poor solubility in the solvent used (acetonitrile).

Importantly, a specific base was used with each coupling agent, aimed at promoting their associated reaction mechanism. However, this parameter was not varied, and it could also be subjected to further optimisation (type and concentration). In addition, the analytical results also commonly showed the appearance of an anhydride species formed by the carboxylic acid (promoted by the coupling agent), which competed with the formation of the product molecules during short reaction times.^38^ This was one of the many possible side reactions identified, and the coupling agent may confer preference of one route over the others. All these factors combined may have also influenced the relative low conversion to product obtained for some reactions.

### 2.5 Success rate of the library

Table 1 shows the relative success rate of the library under different screening conditions. For the 25-reaction library, the success rate could be as low as 16% when using a single synthesis reaction condition *e.g.* using T3P as coupling agent under soft conditions. This result could have been obtained when attempting the library without prior knowledge of the chemistry, achieving very limited success. The selection of the best possible coupling agent at most favourable physical conditions (*i.e.* similar to what can be suggested by a very experienced synthetic chemist) delivered up to a 72% success rate, thus leaving ∼30% of the library unavailable for biological testing and biasing future candidate structures. A modest improvement could be obtained if screening a range of physical conditions (SNOBFIT or DoE), but without modifying the coupling agent delivering up to ∼76% success. Finally, 100% of the desired amides could be successfully synthesised by exploring both the physical conditions and the coupling agent.

Library success rate under different screening conditions

**Table:** 

Single physical condition, single coupling agent	HATU		PyCIU		TCFH		T3P	
	SRxn^a^	LS^a^	SRxn^a^	LS^b^	SRxn^a^	LS^b^	SRxn^a^	LS^b^
Soft (120 s, 50 °C)	10	40%	10	40%	9	36%	4	16%
Mid (360 s, 120 °C)	11	44%	17	68%	16	64%	8	32%
Harsh (600 s, 200 °C)	15	60%	18	72%	17	68%	7	28%

a SRxn: number of successfully synthesised amides, calculated as those with the product peak area at UV 254 nm > 10% of total chromatogram area.

b LS: library success rate (%), calculated as the percentage of the 25 possible amides synthesised (with HPLC product peak area >10%).

**Table:** 

Varying physical conditions and single coupling agent	HATU		PyCIU		TCFH		T3P	
	SRxn^a^	LS^a^	SRxn^a^	LS^b^	SRxn^a^	LS^b^	SRxn^a^	LS^b^
Sequence – 9 physical conditions	18	72%	20	80%	19	76%	11	44%

**Table:** 

HT screening	Successful reactions (SRxn^a^)	Library success rate (LS^b^)
9 physical conditions, 4 coupling agents	25	100%

In this case, the best conditions for each reaction can be inferred from a full screening, subsequently being able to repeat the specific best synthesis points focused on recovery.

The methodology proposed shows an increase in the reaction success rate, which is a critical objective for initial DMTA iterations. The experimental framework achieved this by significantly shortening the experimental turnover times, as well as controlling the heating-cooling rates of the reactor that allowed a brief downtime between experiments. Consequently, the total time to perform an exploratory library synthesis, including the access to a considerably larger pool of reaction conditions, was similar to the time required to run these reactions in batch and under a single condition. The approach facilitated the synthesis of the target molecules at more favourable conditions, pushing the success rate from 16–40% achieved under fixed conditions, up to 100% by applying the high-throughput screening.

### 2.6 Machine learning modelling and validation

A feed forward neural network (FFNN) model consisting of 1–3 hidden layers (see Materials and methods and ESI†) based on the temporal dataset only, provided relatively good predictive performance. We compared this model to a series of models based on a random assignment of the labels, minimizing the risk that a random pick can predict as well as the best model (see ESI†). The model features were based on the reaction conditions and the acid and amine structural information. The cross-validation study also revealed that high performing models can be observed in the cases where the training and test sets used for validation were built on random selection. The ‘leave-one-amine-out’ models present lower performance, which was expected since the amine set was small. Performance metrics such ROCS (Receiver Operating Characteristic Curve) and ‘precision’ chosen to select the best final model which displayed significantly more robust prediction when analysing predictions on the cross-validation dataset. The ‘precision’ has been preferred over the ‘recall’ in order to maximize the ratio of successful experiments over the unsuccessful ones. Critically, the model based on the combined fingerprint, condition and molecular properties sets showed more consistent performance throughout the different cross-validation ‘hold-on’ test sets and good performance in predicting the temporal test set as discussed hereafter. Detailed analysis around the trends in prediction for the different amines and cross-validation studies that guided the selection of the most promising model is available in the ESI.†

**Fig. 5:**
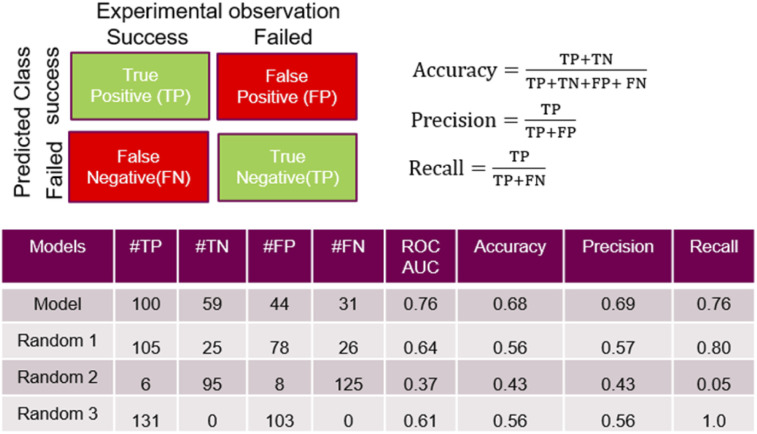
Performance measures for the model and random models. TP (true positive) and TN (true negative) correspond to successful and failed experiments that were correctly predicted by the model, respectively; oppositely, FP (false positive) and FN (false negative) correspond relate to wrong outcome predictions. The ‘precision’ is the ratio of the relevant instance (here, the true positive) among all the retrieved instances (true or false positive). The ‘recall’ is the fraction of relevant instances that were retrieved. The ‘accuracy’ can be seen as a measure of how often the model make a good prediction (whatever it is a successful or a failed experiment).

Some quality measurements using the best selected model in predicting the temporal test set are presented in Fig. 5 (see ESI† for additional metrics and the descriptions of the model performance evaluations used). To rule out the possibility that a random model based on the whole 936 sets could predict as accurately the temporal test set as the model, Fig. 5 also depicts the predicted data based on 3 randomized models (same strategy as applied during the cross-validation study).

**Fig. 6:**
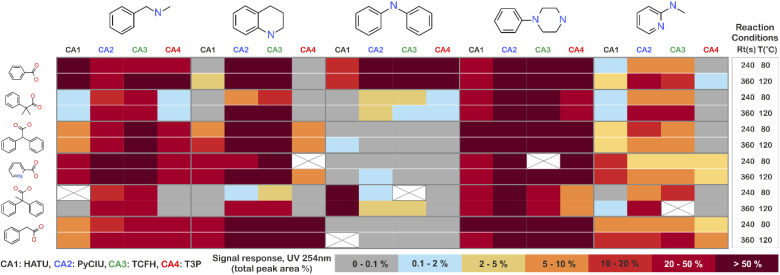
Experimental results obtained for a second amide library synthesised for external model cross-validation. Six carboxylic acids (Scheme 1, A1 to A6) were combined with five new amines (Scheme 1, B6 to B10), using the four coupling agents (Scheme 1, CA1 to CA4) and under two different reaction conditions totalling 240 individual reactions. Colours indicate the relative abundance of the target molecule (UV peak area of the target molecule relative to the total chromatogram area (%), measured at 254 nm). White and crossed boxes indicate absence of analytical data due to instrument failure.

Experimental validation of the machine learning models was achieved through an additional 30-product reaction library containing a new group of amines (Scheme 1, B6 to B10) with the previous carboxylic acids (A1 to A5) plus a new carboxylic acid (A6) was synthesised in high-throughput mode. Two physical conditions (within the ‘mid’ reactions conditions) were used, varying the same initial four coupling agents. A heatmap with the experimental results obtained is shown in Fig. 6. In these circumstances, the success rate achieved (when the product peak area >10%) was 87%. For this library, the group of amines reacted differed to the initial group for which the coupling agents were selected. This was done to illustrate how different experimental and modelling results could deviate from a small modification of the chemistry.

### 2.7 Machine learning prediction of optimal conditions

**Fig. 7:**
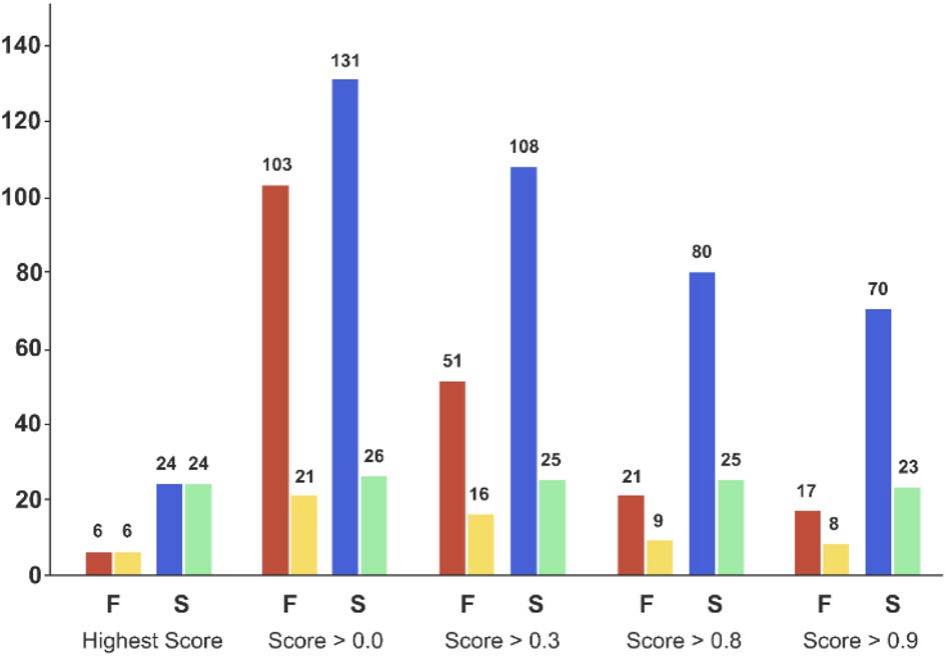
Bar plot of the number of experiments and products with respect to the model score and the reaction outcome in the ‘temporal test set’. On the *X*-axis, ‘Highest Score’ was computed for each product and corresponded to the experiment which obtained the highest model score for a given product. ‘Score > 0.0’ means that no threshold has been applied on the model score (it considers all the experiments). ‘Score > 0.3’ to ‘Score > 0.9’, means that only the experiments for which the model reports a score equal or higher than the corresponding threshold value were considered. Red bars correspond to the number of experiments that failed, and blue bars the number of experiments that succeeded experimentally. Yellow bars correspond to the number of unique products that were targeted by experiments that subsequently failed, while green bars correspond to the number of unique products that succeeded reaction. Experimental results obtained for a second amide library synthesised for external model cross-validation. Six carboxylic acids (Scheme 1, A1 to A6) were combined with five new amines (Scheme 1, B6 to B10), using the four coupling agents (Scheme 1, CA1 to CA4) and under two different reaction conditions totalling 240 individual reactions. Colours indicate the relative abundance of the target molecule (UV peak area of the target molecule relative to the total chromatogram area (%), measured at 254 nm). White and crossed boxes indicate absence of analytical data due to instrument failure.

Applying the chosen model based on the temporal set can significantly reduce the number of experiments required to successfully synthesise unseen candidates – the critical goal of a DMTA cycle. As we are limited by the data in the validation set, we limited prediction to the two ‘mid’ reaction conditions and selection of coupling agent to achieve successful synthesis of each of the unseen 30 combinations. This enables us to illustrate the possibility of using the current model to reduce failure and increase the experimental productivity, Fig. 7 displays the number of experiments and products with respect to the model classification score (ranging from 0 to 1) and the reaction outcome. Clearly, the number of experiments decline as the classification score increases (Fig. 7, red and blue bars) while the number of products in successful reaction tend to remain constant (Fig. 7, yellow and green bars). In other words, selecting the experiments with higher scores increase the relative frequency of the successful experiments compared to the failed reactions. For example, in the case where all the 234 experiments are considered (score > 0.0 bars) will lead to the synthesis of 26 products (*e.g.* the maximum of product synthesis achieved in the second campaign), increasing the score steadily reduces the number of experiments to run (red and blue bars), and more proportionally the ones which failed experimentally (red bars). Applying a classification score of 0.8 reduces by approximately half the number of experiments (from 234 to 101), with only one product not being synthesised (25 instead of 26, see green bar in Fig. 7). Excitingly, running only the experiments suggested that have obtained the highest classification score per product could deliver 24 (92% of the successful syntheses achieved in 2nd campaign) products with only 30 experiments. In summary, the current model score can already be used to substantially reduce the number of experiments required, however enabling access to the full experimental space (*i.e.* 2 → 9 reaction conditions) would be preferred in order to increase the success rate higher.

Finally, model improvements could likely be achieved by considering other experimental or more sophisticated quantum mechanics molecular descriptors. In our case the model performance to reproduce the first experiments (cross-validation study) and to predict experiments based on new reactants presenting similar reactive environment showed that the models based on common and rather computationally inexpensive molecular descriptors already captured accordingly the structural features needed to successfully predict the conditions required to synthesise product accurately. In addition, to further increase predictability experimental datasets should be expanded, for example the amine diversity in the training data in this work would expand predictability significantly.

## 3 Conclusions

In this work, we demonstrated the use of stopped-flow synthesis as an alternative to traditional batch and flow chemistry methods to synthesise diversity-oriented libraries. The stopped-flow reactor concept was deployed for library synthesis and focussed on building scaffold diversity, by using the reactor in high-throughput mode to create a map of the chemistry and the underlying reaction conditions required to complete the transformations. This led to an understanding of the optimum synthetic conditions, increasing the success rate of an initial library from 16–40% (obtained under a fixed single condition) up to 100% when applying the high-throughput screening method.

For both applications, the reduction in the amount of reagents required to synthesise enough quantities for bioassays is a key advantage compared to traditional approaches (∼90% reduction compared to continuous flow), making the system significantly more sustainable compared with other platforms operating in fully continuous mode. Notably, the experimental framework deployed for the stopped-flow reactor can be replicated independent of the hardware available, which could prove valuable in the future by enabling direct comparisons of chemical reactions obtained from independent platforms. Significantly shorter experimental turnover times were achieved by controlling the heating-cooling rates of the reactor, enabling a fast transition between experiments and consequently easing synthesis bottlenecks.

Finally, the experimental results were used to build a machine learning model able to predict the synthesis conditions with 92% accuracy on first attempt, which was validated using external experimental information. It is expected that the systematic gathering of data (as described in this study) will enhance the predictability of synthesis conditions made by machine learning methods.

## 4 Materials and methods

### 4.1 Stopped-flow reactor

**Fig. 8:**
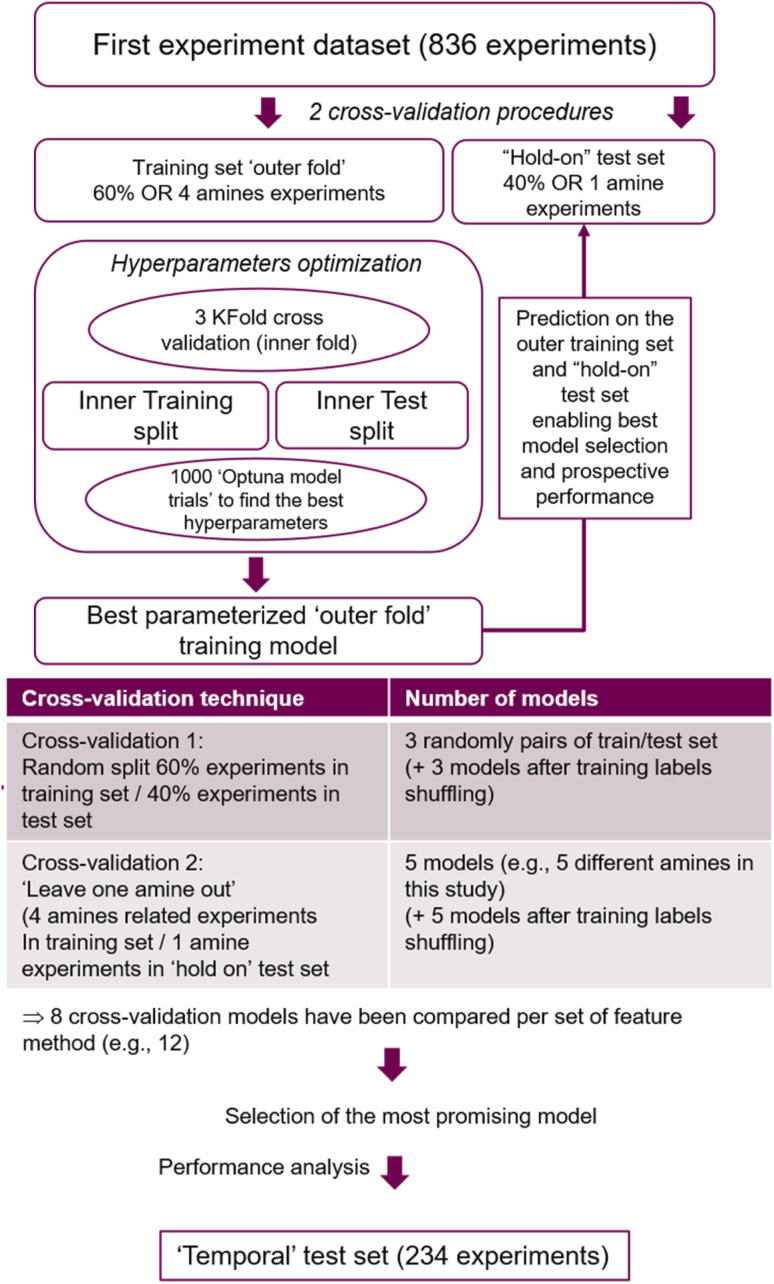
Flow chart of the best model selection strategy applied to the 12 different features set used in this study.

The reactor used was a 1000 μL coil (0.04 mm diameter) twisted around and in full contact with an aluminium cylinder block (Fig. 1b, 5–8). The inlet of the reactor was connected to a cross piece (acting as a mixer) linked to each of the sampling loops lines, while the reactor outlet was connected to a stainless-steel back pressure regulator (BPR), equipped with a cartridge for 750 psi (Fig. 1b, 8). The reactor temperature was externally controlled by temperature controller Cal 9300 (Eurotherm Ltd, UK), connected to a k-type thermocouple and a pair of heating cartridges, all elements embedded into the centre of the aluminium block (Fig. 1b, 5). In addition, fast reactor cooling between experiments was achieved by using a copper pipe twisted around the external aluminium cylinder (in contact with the inner reactor coil), connected to a cooling water supply which was automatically triggered at the end of each reaction (additional details are provided in the ESI†).

### 4.2 Integration of stopped-flow reactor into a high-throughput platform

The stopped-flow reactor was integrated into a high-throughput platform comprised of four hardware modules centrally controlled (Fig. 1b shows a diagram of the system).

#### 4.2.1 Sample preparation

A Gilson 215 liquid handler equipped with an internal syringe pump of 1 mL capacity was used to sample from the initial stock vials and direct the reagent to one of the three sampling loops available, by using a 10-port multiposition valve (Valco Instruments Co, USA). Cleaning cycles for the needle and sample lines were implemented using available valve ports.

#### 4.2.2 Injection

Three sampling loops were arranged in parallel using three individual 2-position 6-port sampling valves model Rheodyne MXP7920 (IDEX Health & Science LLC, USA). The ports configuration for each loop was (Fig. 1b): Port 1, connected to the reagent line coming from the multi-selection valve and liquid handler; Port 2, connected to an HPLC pump model Azura 4.1 (Knauer, Germany) equipped with 10 mL pump head and used to pump clean solvent; Ports 3 and 6, connected to a sampling loop of 500 μL of capacity; Port 4, connected to a cross piece linked to the reactor; and Port 5, connected to a waste line. In position A (default), the pumps flush the reactor line with clean solvent while the sampling loops are filled by the liquid handler. Position B is the reaction condition.

#### 4.2.3 Reaction

When instructed by the software, the three valves simultaneously switch to the position B (reaction). In this position, the pumps simultaneously push out the reagents captured in the sampling loops at a constant flow rate of 0.5 mL min^−1^ (total flow rate 1.5 mL min^−1^), flowing towards the reactor (travelling reaction volume of 1.5 mL), and positioning the centre of the reacting volume at the reaction point. When the sample reaches this position, the pumps are stopped, and the system waits for the desired reaction time to complete. When the desired reaction time has elapsed, the pumps are reactivated, moving the reaction volume out of the reactor and passing through the analytical sample points. At the end of the reaction stage, the valves return to the default position (position A) for reloading.

#### 4.2.4 Analytical

An online NIR flow cell (positioned after back pressure regulator) was used to monitor the position of the reaction volume and timing the analytical sampling valves injection. The flow cell was an inline Z type flow cell connected by a pair of fibre optic cables to a MEMS-FPI sensor with integrated light source model 1.7 (Spectral Engines, Finland). After the flow cell, a 2 μl sample was taken from the centre of the reaction volume by using a 2-position 6-port sampling valve model Cheminert (Valco Instruments Co, USA), simultaneously triggering an Infinity 1260 HPLC system (Agilent Technologies Ltd, UK) equipped with a DAD detector, and an Expression-L mass spectrometer (Advion Inc, USA) equipped with an Electrospray Ionization (ESI) chamber.

#### 4.2.5 System control

All the system elements were controlled using a custom-made software application written in Labview 2017 (National Instruments, USA). This software controlled the instrument communication and reaction time sequences, reading the outputs from the analytical instruments and performing the data analysis required. This also communicates with other parallel algorithms implement such as the self-optimisation method (SNOBFIT)^39^ executed using an embedded add-on written in MATLAB R2019a (The MathWorks Inc., USA).

### 4.3 Sample preparation

A 25 compound library was synthesised combining five carboxylic acids (Scheme 1, A1 to A5) with five amines (Scheme 1, B1 to B5; as limiting reagent), using four different coupling agents (Scheme 1, CA1 to CA4; HATU,^40^ PyCIU,^37^ TCFH,^38^ and T3P).^40^ Each reaction sequence required the used of three vials (20 mL each) containing the respective stock solutions (all prepared in acetonitrile), placed on the designated liquid handler trays. The vials were referred as: *vial a* for the acid, *vial b* for the amine, and *vial c* for the coupling agent (Fig. 1c). The specific base selection depended on each of the coupling agents used:

CA1, HATU (*O*-(7-azabenzotriazol-1-yl)-1,1,3,3-tetramethyluronium hexafluorophosphate): *vial a*, acid (0.13 M) premixed with *N*,*N*-diisopropylethylamine (0.35 M); *vial b*, amine (0.1 M) premixed with toluene (0.1 M, used as internal standard); *vial c*, HATU (0.15 M).

CA2, PyCIU (chlorodipyrrolidinocarbenium hexafluorophosphate): *vial a*, acid (0.13 M) premixed with 1-methyl-1*H*-imidazole (0.35 M); *vial b*, amine (0.1 M) premixed with toluene (0.1 M, used as internal standard); *vial c*, PyCIU (0.15 M).

CA3, TCFH (*N*,*N*,*N*′,*N*′-tetramethylchloroformamidinium hexafluorophosphate): *vial a*, acid (0.13 M) premixed with 1-methyl-1*H*-imidazole (0.35 M); *vial b*, amine (0.1 M) premixed with toluene (0.1 M, used as internal standard); *vial c*, TCFH (0.15 M).

CA4, T3P (2-propanephosphonic acid anhydride): *vial a*, acid (0.13 M) premixed with triethylamine (0.35 M); *vial b*, amine (0.1 M) premixed with toluene (0.1 M, used as internal standard); *vial c*, T3P (0.15 M).

For external model validation, a 30-compound library was attempted combining six carboxylic acids (Scheme 1, A1 to A6) with five amines (Scheme 1, B6 to B10; limited reagent), using the same four coupling agents (Scheme 1, CA1 to CA4). The corresponding reaction sequences were performed in the same way as described for the initial 25-compound library.

### 4.4 Analytical methods

HPLC-MS: a generic gradient separation method was performed for all the reactions, starting from an aqueous rich water–acetonitrile mixture (90% water–10% acetonitrile), up to 100% acetonitrile in 5 min, with additional 1.5 min under isocratic conditions at the end of the program (total program time 6.5 min). Both mobile phases contained formic acid (0.1% v/v) to facilitate analyte ionisation and minimise spectral deviations on the DAD signal. All calculations were performed using the HPLC Diode Array Detector (DAD) signal measured at 215 nm and 254 nm.

### 4.5 Machine learning model

A feed-forward neural network (FFNN) model was built using the experimental data obtained from the 25-compound library (836 data points), and its performance in predicting the results obtained from the 30-compound library (234 data points) was evaluated. The machine learning work had two main objectives: first, to assess if a predictive model can be trained on the current small dataset and how it performed in predicting new experiments based on new reactants structures. Secondly, to analyse if and how the current model classification scores could be used to guide future experiments in order to most rapidly generate new compounds. In addition, two different cross-validation strategies were compared; and the influence of using different set of model features has been also investigated.

#### 4.5.1 Modelling strategy

Fig. 8 illustrates the modelling strategy. In this, a series of models were built and evaluated on the first data set (836 experiments) following a cross-validation procedure. The main objective of the cross-validation was to identify which type of model features should be employed to train and to evaluate the ‘true’ predictive power of the model, in order to best predict the second library set (234 data points, also refereed as ‘unseen’ data or the ‘temporal test set’). Two distinct cross-validation strategies were used. In a first case, the dataset was randomly split in two parts, ‘outer training’ and ‘hold-on test’ sets, containing 60% and 40% of the experiments respectively. Data randomization was used to detect any bias in the distribution of the experiments between the training and test sets, finding 3 random splits sufficient. In the second cross-validation procedure, datasets used to train the models were built following a ‘leave-one-amine-out’ method, generating 5 different training sets using all the experiments corresponding to 4 amines (out of 5) as a base, while the remaining ‘out-amine’ experiments were left in the ‘hold-on’ test set. Finally, for both cases the temporal test set was not used in any of the model building, optimization, calculation and selection, only used at the final performance testing stage to avoid overfitting.

The procedure displayed in Fig. 8 was run 12 times, corresponding to 12 different feature sets covered. Feature based models were compared on their performance predicting the ‘hold on’ test sets derived from the two cross-validation strategies. From these, the model presenting the best overall ‘hold on’ test set performance was identified. Subsequently, its feature method and optimized hyperparameters were considered to train the final model based on the whole 836 experiments dataset, and its accuracy on predicting the ‘temporal test set’ was established.

#### 4.5.2 ‘Two classes classification’ based feed-forward neural network model

The feed-forward neural network (FFNN) models developed were implemented in python using the Keras package (v 2.3.1). A simplified version of the code is available in GitHub as a Jupiter Notebook.^41^ As the signal response vary differently between different products, it cannot be used to build a regression-based model. Instead, experiments were categorized into two groups to enable building a ‘two classes’ classification model. The two groups correspond to successful reactions (labelled as ‘successful’), corresponding to the experiments with a signal response (peak chromatogram area) equal or higher than 10%, and failed reactions (labelled as ‘failed’), corresponding to the experiments with a signal response (peak chromatogram area) lower than 10%. This cut-off value was based on an experimental rule of thumb from which the recovery of the product is feasible.

#### 4.5.3 Model architecture and hyperparameters optimization

The FFNN model architecture is based on a certain number of parameters called ‘hyperparameters’ *e.g.* number of hidden layers, nodes, number of training cycles or epoch; which remain constant during the model training process. In order to find the optimal combination of hyperparameters that maximize the performance of each model, a series of different setups were evaluated, performed using an Optuna implementation (detailed model characteristics and the hyperparameter search space can be found in the ESI†).^42^ For this, within an inner 3 K-fold cross-validation procedure, 1000 model trials corresponding to 1000 hyperparameter sets were built on the inner training set, and subsequently evaluated using the ‘binary_accuracy’ metric for predicting the inner test set (ESI, Fig. S5.1†). This accuracy measurement was chosen as it turned out that the two classes are properly balanced. Additionally, in order to fairly evaluate the predictive power of the model, different model performance measurements were compared to those derived from random predictions. For this, ‘random models’ were built by randomizing the labels in the training set and going through the same hyperparameter optimization process than the ‘true’ models based on non-shuffled data. In summary, for each set of model features (discussed below), 16 ‘best hyperparameters’ models were built (8 without shuffling the labels, and 8 with label shuffling) on the basis of 3 different random splits (60/40%), and 5 ‘leave-one-amine-out’ dataset stratification (ESI, Fig. S5.1†).

#### 4.5.4 Model features

Each model feature encodes different aspects of the experimental information. The developed models were built using a single or a combination of feature types (see ESI†), from which the most relevant included: ‘reaction fingerprint’, obtained by subtracting from the product molecular fingerprint the reactant fingerprints;^43^ and ‘product fingerprint’, used to compensate for structural differences between the reactants that were explicitly removed during the reaction fingerprint subtraction. Reaction and product fingerprints were obtained using Morgan fingerprint as implemented in RDKit (radius set to 3) or this fingerprint concatenated with the RDKit chemical fingerprint.^44^

The reaction condition relying on the choice of the coupling agent, reaction time and temperature information was implemented as three ‘one hot vector’ presenting a number of bits corresponding to the number of possibilities for each condition components. Thus, each experiment condition is described by a feature set of 14 bits (*e.g.* 4 coupling agents, 5 different temperatures and times). Also, some tested models included computed reactant molecular properties, corresponding to a set of descriptors for physiochemical properties which has proved efficient at AstraZeneca (code used to calculate those descriptors cannot be shared, but computed properties are provided in the ESI†).^45^ Finally, p*K*_a1_ to p*K*_a4_ were calculated using ACDPK Labs software and used in combination with the physiochemical properties for some models.

## Data availability

The datasets supporting this article have been uploaded as part of the ESI.†

## Author contributions

CA, ADC, TK and RAB wrote the manuscript, CA performed the experiments and developed the control software, CC, TK, JM and SS provided the experimental and modelling methodologies, CA and TK performed the visualization of data. RAB, CPG and MK conceptualized the research and supervised the research.

## Conflicts of interest

There are no conflicts to declare.

## Supplementary Material

SC-013-D2SC03016K-s001

SC-013-D2SC03016K-s002

SC-013-D2SC03016K-s003

SC-013-D2SC03016K-s004

SC-013-D2SC03016K-s005

SC-013-D2SC03016K-s006

SC-013-D2SC03016K-s007

SC-013-D2SC03016K-s008

SC-013-D2SC03016K-s009

SC-013-D2SC03016K-s010

SC-013-D2SC03016K-s011

SC-013-D2SC03016K-s012

SC-013-D2SC03016K-s013

SC-013-D2SC03016K-s014
