# Supplementary material for: Automated stopped-flow library synthesis for rapid optimisation and machine learning directed experimentation
Source: Chem Sci. 2022 Sep 13;13(41):12087–99. doi: 10.1039/d2sc03016k (PMC9601405; doi:10.1039/d2sc03016k)
Supplement: SC-013-D2SC03016K-s014 [file SC-013-D2SC03016K-s014.pdf]

Electronic Supplementary Material (ESI) for Chemical Science.
This journal is © The Royal Society of Chemistry 2022

Automated stopped-flow library synthesis for rapid optimisation and machine learning directed experimentation

Claudio Avila,[a,d] Carlo Cassani,[b] Thierry Kogej,[c] Javier Mazuela,[c] Sunil Sarda,[a] Adam D. Clayton,[d] Michael Kossenjans,[b] Clive Green[a] and Richard Bourne[d]

[a]*Sample Management, Discovery Sciences, BioPharmaceuticals R&D, AstraZeneca, Cambridge, CB4 0WG, UK.*

[b]*Research and Early Development, Medicinal Chemistry Respiratory & Immunology, Göteborg, Sweden.*

[c]*MolecularAI, Discovery Sciences, R&D, AstraZeneca, Göteborg, Sweden.*

[d]*School of Chemical and Process Engineering, University of Leeds, Leeds, LS2 9JT, UK.*

S1. Continuous flow limitations

Despite the benefits, continuous flow chemistry still shows some disadvantages for medicinal chemistry such as the synthesis scale (relatively high), requiring to pump large reagent volumes to achieve steady state conditions [1], with unnecessary environmental and economic costs (e.g. when using hazardous or expensive reagents) [2]. For small volumes (microreactors), the use of dedicated and expensive equipment is necessary to achieve low flow rates required for relatively long residence times [3]. Figure S1 illustrate some of these limitations.

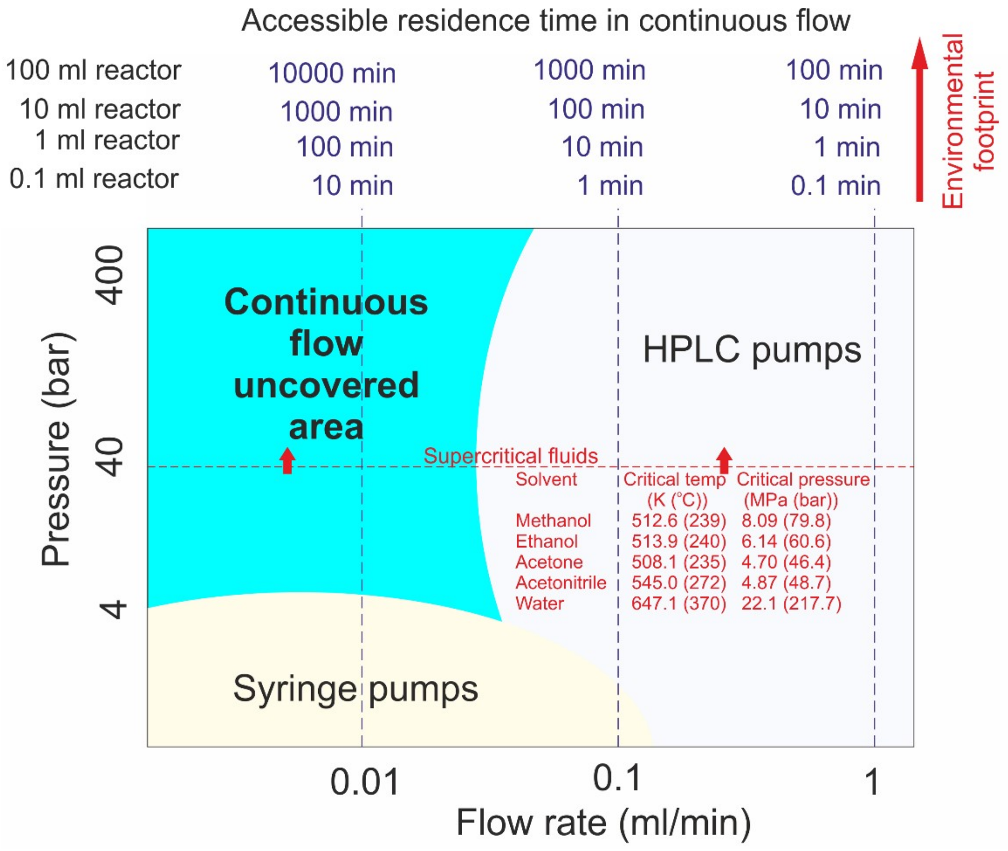

Figure S1. Limitations of continuous flow chemistry of interest for drug discovery.

S2. Stopped-flow reactor operation

The reactor used was a 1000 µL coil (0.04 mm diameter) twisted around and in full contact of an aluminium cylinder block (Figure 1b, 5-8). The inlet of the reactor was connected to cross piece linked to each of the sampling loops lines, while the reactor outlet was connected to a stainless-steel back pressure regulator (BPR), equipped with a cartridge for 750 psi (Figure 1b, 8). The reactor temperature was externally controlled by a Eurotherm temperature controller, connected to a k-type thermocouple and heated up by a pair of cylindrical heating cartridges, all elements embedded into the centre of the aluminium block (Figure 1b, 5). In addition, fast reactor cooling between experiments was achieved by using a cooper pipe twisted around the external aluminium cylinder (in contact with the inner reactor coil), connecting to a cooling water supply which was automatically triggered at the end of each reaction. Finally, the reactor block was thermally insulated with a cotton jacket covering all external parts.

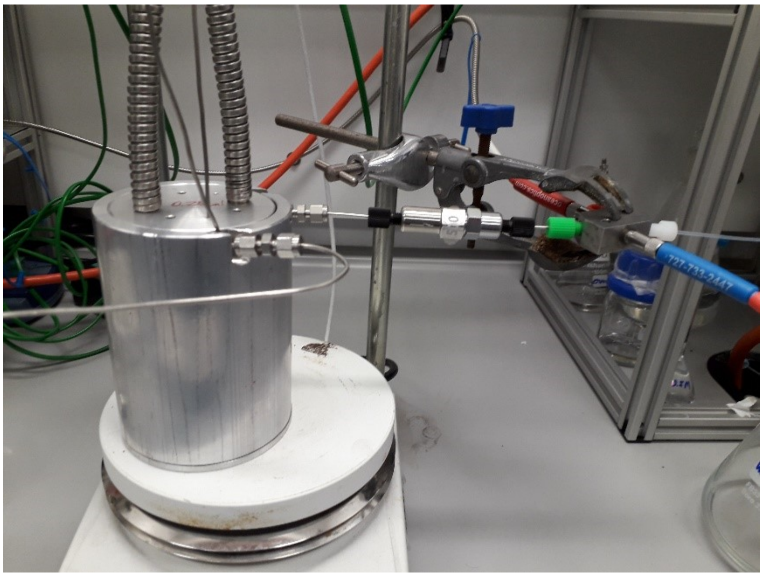

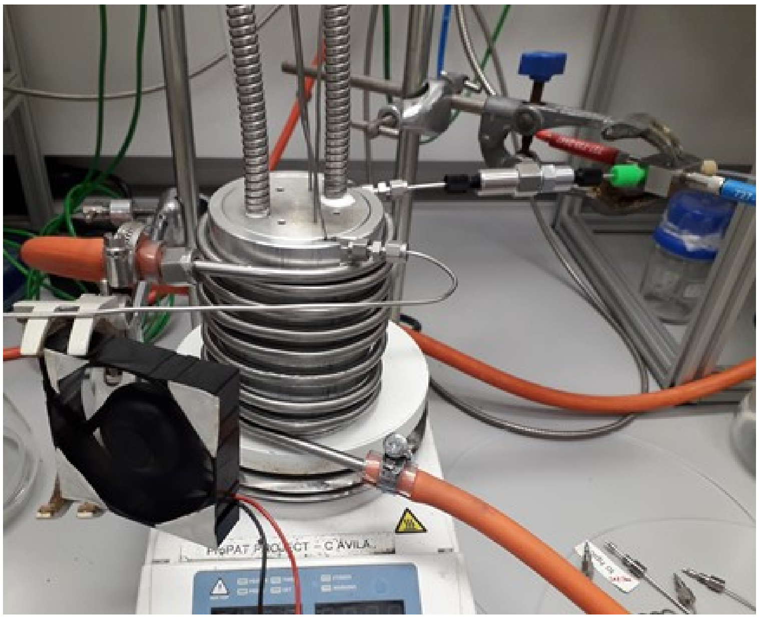

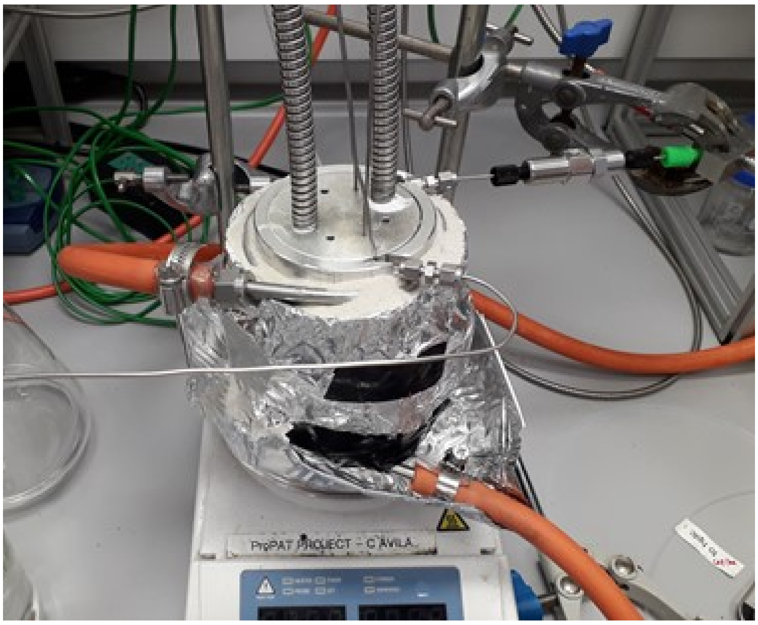
ProPAT PROJECT - C AVILA

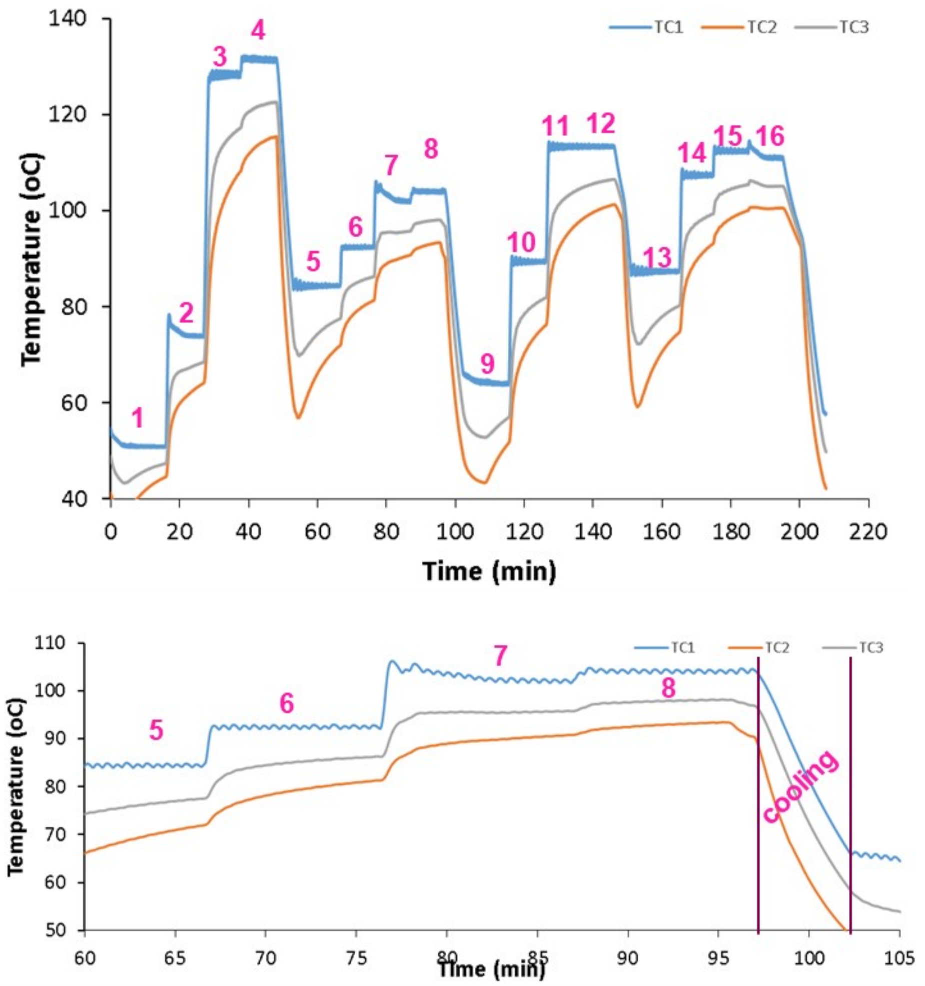

Figure S2. Photos of reactor with heating elements and cooling jacket. Graphs showing temperature profiles of subsequent experiments.

S3. SNOBIFT Optimisation

The first approach applied was searching for the optimum reaction conditions i.e. temperature and reaction time, using an automated self-optimisation algorithm. In this case, an iterative single-objective self-optimisation method was programmed (SNOBFIT [37]; Figure S3, a), minimising the ratio of the internal standard to the product (using their respective HPLC UV response peak signal areas), when varying temperature and reaction time. A summary of the results obtained for two amide coupling reactions is discussed (Figure S3, b and e). For these reactions, the self-optimisation method was successful to identify the best reaction conditions, when chemical variables were kept constant (Figure S3, c and f). The HPLC-MS data provided an in-depth visualisation of the interactions between species (products and side-products), revealing their influence in the reaction path to synthesise the target molecule. These interactions were aligned with the optimum reaction conditions identified from the self-optimisation (Figure S3, d and g).

In the library synthesis context, the experimental process driven by the algorithm was a relative time-consuming task, requiring a large number of experiments i.e. ~45 conditions tested for each reaction requiring a total of 10 hours (similar to other self-optimisation algorithms implemented [28]). An unrestricted large number of experiments can be particularly problematic when the search diverges e.g. when the reaction does not proceed under any circumstance. In addition, the random exploration path differs from reaction to reaction, making it difficult to compare and frustrating the identification and modelling of global underlying trends of the whole library. Finally, the algorithm implementation also required the use of an internal standard to calculate the relative increase of the target molecule, and prior knowledge of the chromatographic retention characteristics of the target product. Consequently, we found that applying self-optimisation methods more applicable when

towards the end of the DMTA cycle, at the hit-to-lead optimisation stage when libraries are small.

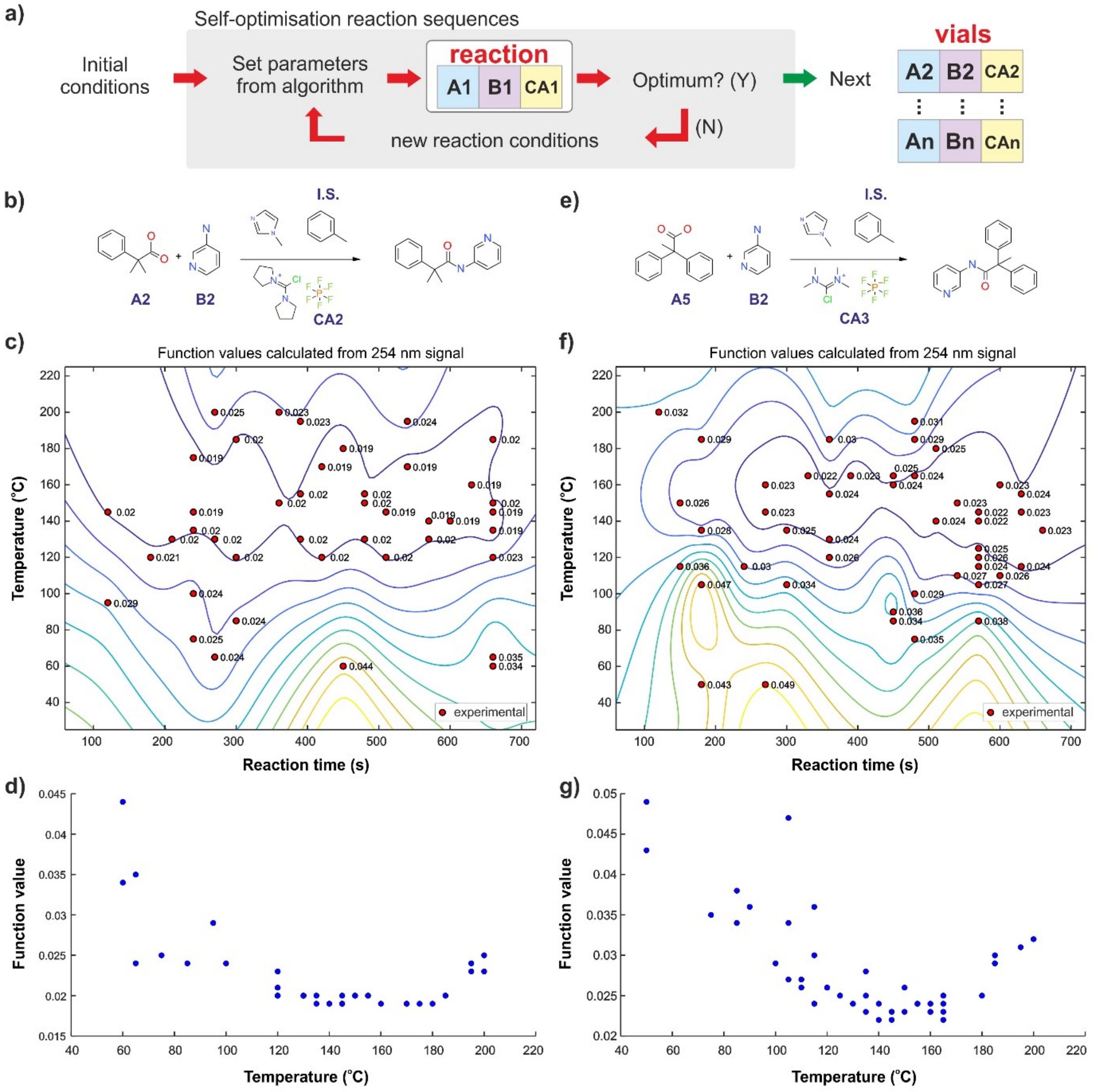

Figure S3. Automated self-optimisation reaction sequences driven by SNOBFIT algorithm (a). Two amide coupling reactions subjected to a self-optimisation algorithm (b and e), designed to identify the best reaction conditions (temperature and reaction time) for the targeted product molecule. (c) and (f) illustrate their respective contour plots, obtained by minimising a function value (the ratio between the internal standard to the product peak areas, calculated from the HPLC DAD 254 nm signal). In both cases, the maximum yield was obtained when the target

molecule was competing with the generation of side-products under a strong temperature dependency (d and g), and with minimal effect of the reaction time (all Rt points are plotted).

S4. Function values for optimisation

For the self-optimisation (Figure S3), the ratio between internal standard to the desired product was calculated using the HPLC signal at 254 nm. For each reaction the acquired data is presented in table S4.1 and S4.2 respectively.

Table S4.1. Sequence of experimental points driven by SNOBFIT algorithm, obtained for the Reaction 7 using PyCIU as the coupling agent.

Experimental point	Function value (I.S./COMP)	Reaction time (s)	Temperature (^oC)
1	0.034	660	60
2	0.020	240	135
3	0.020	120	145
4	0.019	450	180
5	0.020	660	185
6	0.024	540	195
7	0.044	450	60
8	0.035	660	65
9	0.029	120	95
10	0.019	570	140
11	0.023	390	195
12	0.025	270	200
13	0.023	660	120
14	0.020	300	120
15	0.020	510	120
16	0.019	660	135
17	0.020	300	185
18	0.023	360	200
19	0.024	300	85
20	0.021	180	120
21	0.020	570	130
22	0.020	390	130
23	0.019	240	145
24	0.020	660	150
25	0.024	240	100
26	0.020	420	120
27	0.020	270	130
28	0.019	600	140
29	0.020	360	150
30	0.019	540	170
31	0.025	240	75
32	0.020	480	130
33	0.020	210	130
34	0.020	480	150
35	0.020	480	155
36	0.019	240	175
37	0.024	270	65
38	0.019	510	145

39	0.019	660	145
40	0.020	390	155
41	0.019	630	160
42	0.019	420	170

Table S4.2. Sequence of experimental points driven by SNOBFIT algorithm, obtained for the Reaction 10 using TCFH as the coupling agent.

Experimental point	Function value (I.S./COMP)	Reaction time (s)	Temperature (°C)
1	0.2216	450	50
2	0.1250	240	65
3	0.0375	570	85
4	0.0281	180	135
5	0.0234	390	165
6	0.0323	120	200
7	0.0429	180	50
8	0.0266	570	105
9	0.0297	240	115
10	0.0243	570	115
11	0.0355	150	115
12	0.0241	480	165
13	0.0486	270	50
14	0.0241	630	115
15	0.0247	300	135
16	0.0219	570	145
17	0.0251	510	180
18	0.0314	480	195
19	0.0290	480	100
20	0.0257	360	120
21	0.0238	360	130
22	0.0221	570	140
23	0.0231	270	145
24	0.0229	600	160
25	0.0340	300	105
26	0.0230	660	135
27	0.0229	540	150
28	0.0257	150	150
29	0.0223	330	165
30	injection missed	injection missed	injection missed
31	0.0471	180	105
32	0.0256	570	120
33	0.0235	630	145
34	0.0236	450	160
35	0.0290	480	185
36	0.0289	180	185
37	0.0352	480	75
38	0.0268	540	110
39	0.0249	570	125
40	0.0234	270	160
41	0.0250	450	165
42	0.0296	360	185

43	0.0339	450	85
44	0.0365	450	90
45	0.0263	600	110
46	0.0244	510	140
47	0.0236	360	155
48	0.0244	630	155

S5. Machine learning modelling

S5.1 Feed-Forward Neural Net Model architecture

The feed forward neural network (FFNN) consists in 1-3 hidden layers (will be set by the hyperparameters optimization), see "def build_keras_model" in the provided code in Github (github.com/MolecularAI/HTE_Publication_Avila_et_al). 'Dropout' is applied at each layer. The output layer consists in sigmoid activation function. The 'binary_accuracy' has been chosen as metric to access the performance as the two classes are balanced. The learning function is the 'rmsprop' (in some initial testing, adam optimizer has been used but did not lead to significantly different results).

S5.2 Hyperparameters optimization

Table S5.1. Hyperparameters that have been used in this study.

**Feed-forward Neural Network Hyperparameters tuned in this study**	**List of values**
batch_size	One of [32, 64]
learning rate	Loguniform values between (0.00001, 0.005)
numepochs	One of [10,15,20,30]
num_hidden_layers	One of [1, 2, 3]
dropout	Uniform between (0.0, 0.9)
hidden_size	One of [128, 256, 512, 1024, 2048] for models with feature vectors of ~2048 bits size<br>One of [128, 256, 512, 1024] for models with feature vectors of ~1024 bits size<br>One of [4, 8 ,16] for models with feature vectors

	of 14 bits size<br>One of [32, 64, 128, 256] for models with feature vectors of ~200 bits size

S5.3 Model features

Table S5.2. Model features that have been used in this study.

Model number	Reaction Fingerprint (number of bits)	Product fingerprint (number of bits)	Oeselma + ACD labs pKa (number of values)	Fingerprint types	Coupling agents, temperatures and times (see Figure) (number of bits)	Total number of features (features length)
#1	0 (no considered)	0	0	-	14	14
#2	0	0	196	-	14	210
#3	1024	0	0	ECFP6	14	1038
#4	2048	0	0		14	2062
#5	512	512	0		14	1038
#6	1024	1024	0		14	2062
#7	1024 (512 + 512)	0	0	ECFP6+ RDkit fingerprint e.g. half of the bits size for each type	14	1038
#8	2048 (1024+1024)	0	0		14	2062
#9	512 (256+256)	512 (256+256)	0		14	1038
#10	1024 (512 + 512)	1024 (512 + 512)	0		14	2062
#11	512 (256+256)	512 (256+256)	196		14	1234
#12	1024 (512 + 512)	1024 (512 + 512)	196		14	2258

The different fingerprint component (e.g. reaction fingerprint, product fingerprint, oeselma, …) have been defined in the main manuscript. ‘Oeselma’ is an internal AstraZeneca set of versatile molecular descriptors describing a set of physchem properties. Here, we added the pKa computed using ACDPK Labs tool (4 values). For the current modelling, a subset of ‘oeselma’ descriptors (94 descriptors) and pKa have been calculated (4 values per molecules) for the acids and amines, their corresponding values have been concatenated leading to a vector of 196. Scaling the model features matrices have showed to lead to the more consistent results, it was done separately for the training and test set using the ‘np.scale(X_train)’ and ‘np.scale(X_test)’ function available in numpy. The coupling agent, temperature and time have been encoded in 3 separated ‘one hot vector’ as illustrated in Table S5.3.

Table S5.3. Feature bits description corresponding to the set of conditions encoding. For example, experiment made with condition: CA4 at 120 degree Celsius during 360 seconds is encoded as a vector of 14 bits: 0 1 0 0 0 0 1 0 0 0 0 1 0 0

Feature bit	Condition component	Value
Bit 1	Coupling agent - CA2	C1CCN(C1)C(=[N+]2CCCC2)Cl.F[P-](F)(F)(F)(F)F
Bit 2	Coupling agent - CA4	CCCP1(=O)OP(=O)(OP(=O)(O1)CCC)CCC
Bit 3	Coupling agent - CA3	CN(C)C(=[N+](C)C)Cl.F[P-](F)(F)(F)(F)F
Bit 4	Coupling agent- CA1	CN(C)C(=[N+](C)C)On1c2c(cccn2)nn1.F[P-](F)(F)(F)(F)F
Bit 5	Temperature	50
Bit 6	Temperature	80
Bit 7	Temperature	120
Bit 8	Temperature	160
Bit 9	Temperature	200
Bit 10	Time	120
Bit 11	Time	240
Bit 12	Time	360
Bit 13	Time	480
Bit 14	Time	600

S5.4 Cross-validation study results and best model selection

In this section we present the cross-validation study that has been made on the primary set of experiments of 836 data. The followed strategy is described in the main manuscript (see Figure 8). This study aims to select which model best classifies between the “successful” and “failed” reaction among the 836 experiments dataset. Hopefully, its performance will hold on the temporal test which is based on 234 experiments. This evaluation can be used to guide new experiments to enrich the current training dataset and improve the overall performance of the current model.

The average ‘ROC AUC’ score (computed using the roc_auc_score function in scikit-learn) on the 3 cross-validation sets (60% Train/40% Test splits) and 5 ‘one-amine-out’ on the training sets and for the ‘hold-on’ test sets for the different feature models (and corresponding random models based on the same training/test sets, those based on shuffled label training data) are presented at Figure S5.1. The higher the ROC AUC is, the better the performance of the model at distinguishing between the ‘successful’ and ‘failed’ reactions. The analysis discussed below on the average ROC_AUC hold for the different models for which the detailed performances are provided in the excel files (“Model_performances.xlss”).

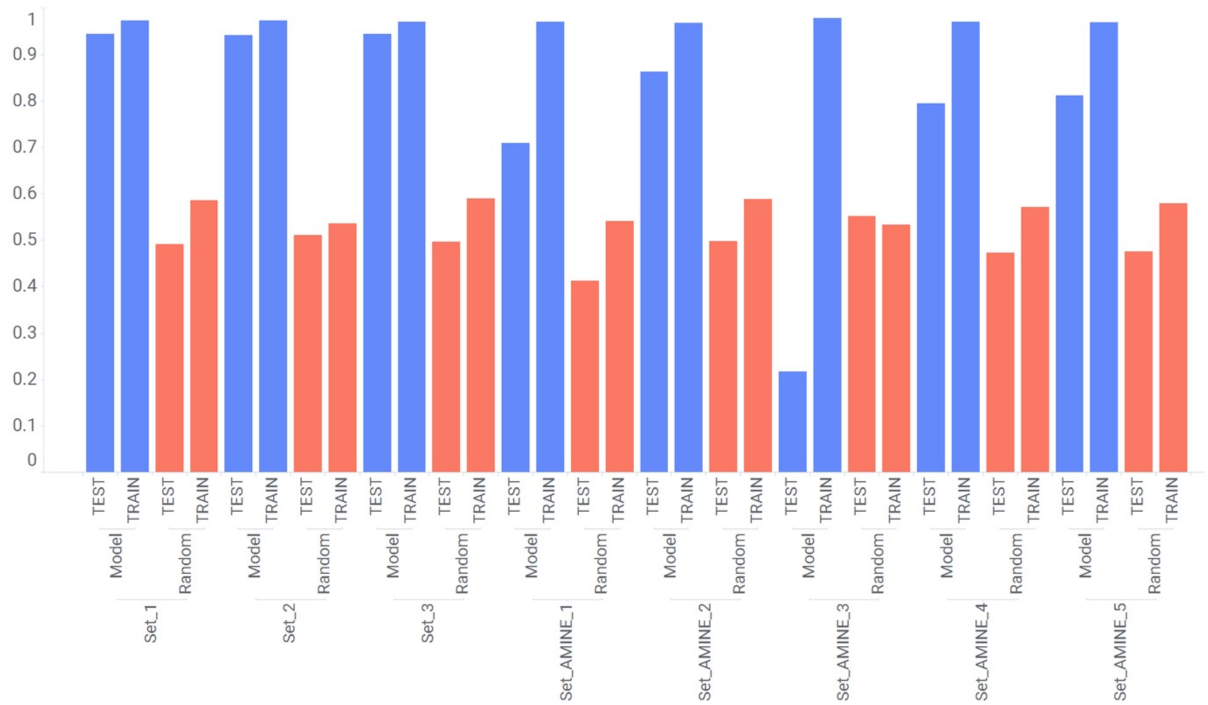

Figure S5.1. Average ROC AUC values on the 12 feature set models with respect to the 8 cross-validation datasets with ("random", red bars) and without ("Model", blue bars) shuffling the training labels. Set_AMINE_1, Set_AMINE_2, … correspond to the 'leave-one-amine-out' cross-validation dataset. Set_1, Set_2, … correspond to the 60%/40% cross-validation datasets. Firstly, the average ROC_AUC for the 'hold-on' test set is very similar than for the ones for the training sets in the case of the three 60/40% cross-validation models (see Set_1, Set_2, and Set_3 in Figure S5.1 and "Model_performance.xlsx" table). In first approximation, this demonstrates that these models do not 'overfit' the training data. Indeed, much poorer performances on the test sets often witness that the models fit the training in a way that it cannot generalize well for external or new data as the test set ones. Secondly, all the 60/40% based models led to better performance than their corresponding random models and for the 'leave-one-amine-out' models. For the latter, the models performances on the training sets are much higher than for the 'hold-on' test sets. This has important consequences. The results on the 'leave-one-amine-out' models revealing that: (i) the amine structure has a great influence on model quality as the one in the test set seems not be well predicted by the ones in the training,

(ii) training model on only randomized data may lead to a overestimation of the model in prediction new experiments which might be based on very different amine types. The amine not present in the Set_AMINE_3 dataset (see structures in Table S5.4) is in overall not well predicted by the different model features types, in fact, the average ROC AUC is lower for the models than the corresponding random models. The presence of the nitro group seems to make the ‘one-out-amine’ in Set_AMINE_3 unique and not well covered by the property profile of the other amines. However, it should be stressed that the training and test set size dataset slightly differ between the different ‘leave one-amine-out’ as all experiments could be collected Table S5.4 also show the number of training and ‘on-hold’ test set data points for the different datasets.

Table S5.4. Structures of the amines that have been considered in the ‘on hold’ test set for the 5 different Set_AMINE_1, Set_AMINE_2, … model datasets as well as the number of data points for the different training and test sets.

# data points	Set_AMINE_1	Set_AMINE_2	Set_AMINE_3	Set_AMINE_4	Set_AMINE_5
	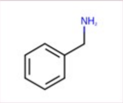	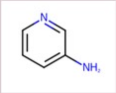	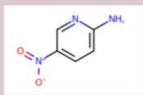	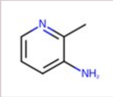	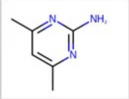
Training set	661	659	695	661	668
Test set	175	177	141	175	168

The ROC AUC gave useful information about the modelling quality but as all the models seem to perform similarly on this score, other quality measure have been used to select the best model to be applied on the temporal test set. This measures is the ‘precision’ (defined in the main manuscript, Figure 5). This values is strongly correlated to the ROC AUC score but maximizes it could help to reduce the number of failed experiments.

Thorough analysis of the performance results presented in “Model_performance.xlsx” showed that the performance differences between many models are subtle. Reassuringly, the model

based on only the conditions as features (Model #1, see Model_performance.xlsx) presents the weakest performance compared to model implying the molecular structures in different manners. We observe that model #11 is among the model set up leading to, overall, high ROC_AUC and precision. The fact that its features set is made of different components such the reaction fingerprint, the product fingerprint and physico-chemical properties made us believe that it has better chance to better predict new data while this was not clearly showed in the cases of the different 'leave-one-amine-out' model though.

As a technical note, the model #11 optimal hyperparameters are: 'batch_size': 32; 'lr': 0.00030645969762385465; 'numepochs': 30; 'num_hidden_layers': 1; 'dropout': 0.0004887962267077486; 'hidden_size': 1024

S5.5 Model analysis

The first observation that can be made is that the performance in predicting the temporal test set is significantly better than the ones from random models which show erratic behaviours. For example, the 'precision' which reflects the capability of the model to find the successful reactions (e.g. true positive) without inflating too many failed reactions (e.g. false positive) is significantly higher for the model versus random models, and it is combined to a high 'recall' which measures how the model is able to retrieve the successful reactions. The latter is higher for the 'Random 1' model but, in this case, the accuracy and precision is poor which translate that the model is overestimating the chance to have productive reaction. However, the performance is lower than the ones observed during the cross-validation study indicating that the current modelling could not generalize enough from the training dataset to lead to highly accurate temporal test prediction. We found understandable that a model based on only 5 different amines and 6 acids would be not be able to predict any amide coupling with high accuracy. Indeed, quite different level of performances have been observed for the various 'leave'one-amine-out' dataset during the cross-validation study and can be explained by the

following analysis. The classification accuracy for the different amines is displayed at Figure S5.2. It can be observed that for 2 out of 5 amines, a significant amount of the related experiments were not well predicted, the diaryl-amine, and the ortho-pyridine amine are related to failed reaction (Figure S5.2, bars marked 'A'). Interestingly, the wrong prediction mainly corresponds to 'false negative' which can be considered less problematic if one wants to maximize the number of produced products. More problematically, the prediction for the experiments using the aryl-piperazine failed in ~34% of the cases, as the model predicts the 16 experiments to fail while they turned to have succeeded.

On the other hand, this type of observations can be considered useful to guide new experiments in order to improve the model. These results clearly demonstrate the need to extent the amine diversity in the training dataset. Nevertheless, some amines, as the benzyl-methyl amine or the 'bi-cyclic' seem to be already well handled by the current model. For example, all failed reactions engaging these amines have been correctly predicted to fail (Figure S5.2, peaks marked 'B'), which demonstrates that the model does not necessary over-estimate the chance for a reaction to succeed, revealing a relatively acceptable 'precision'.

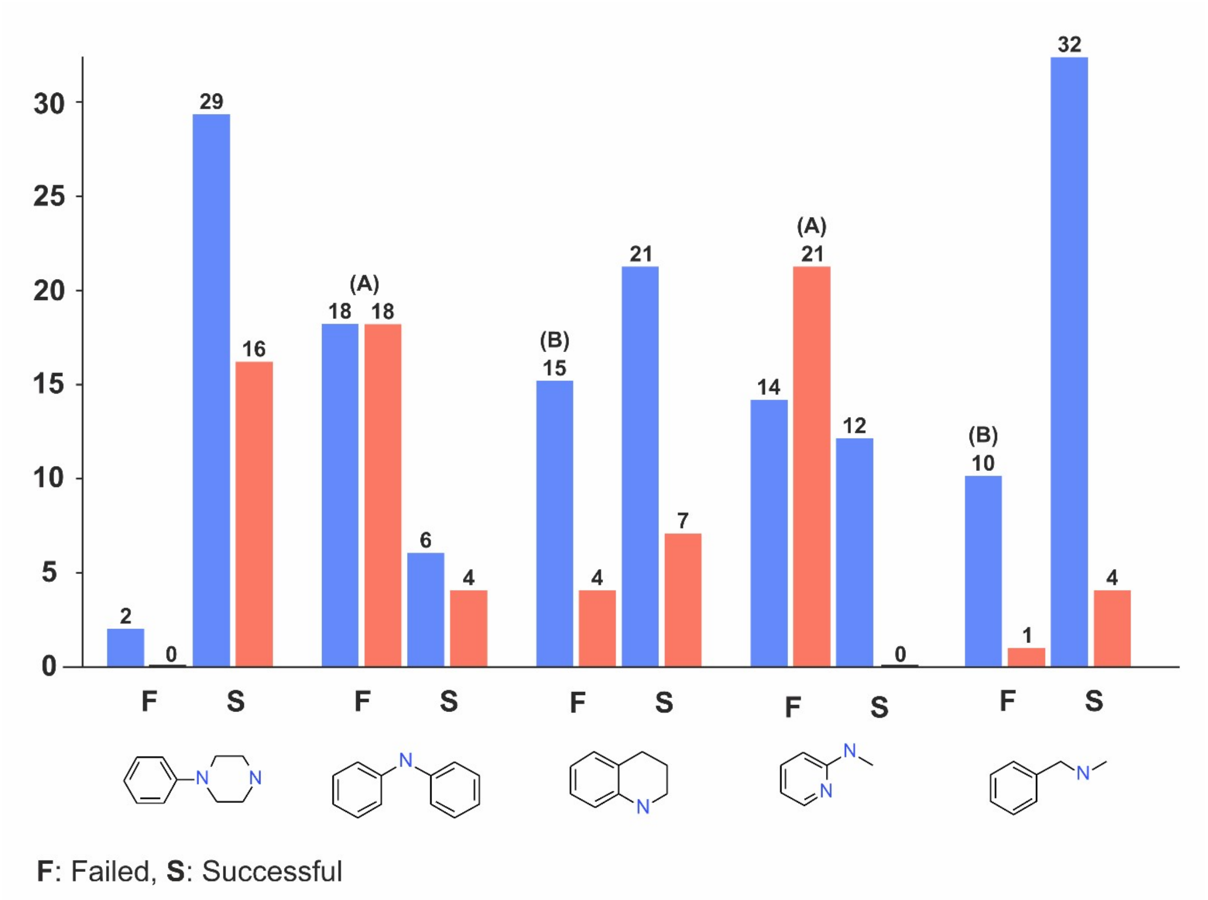

Figure S5.2. Accuracy of the model prediction with respect to the amine. Blue bars represent the number of experiments where the outcome (successful or failed reaction) have been properly predicted by the model. Conversely, red bars represent the number of wrong predictions. The X-axis further informs if the well or badly predicted experiments belongs to successful or failed reactions.

S5.6 Coupling-agent prediction analysis

The coupling agent plays a major role in the amide coupling reaction. Similarly to the amine analysis discussed above, the classification accuracy for the different coupling agents is depicted at Figure S5.3. As for the amine case, some good trends as well as some key learning for improvement can be made. Firstly, there is no coupling agent for which the related experiment are systematically badly predicted by the model. However, failed prediction surpasses good predictions in the case of failed experiments reaction using CA2 meaning the

model had the tendency to overestimate the success of the reaction when this coupling agent is employed (see peak “a” in Figure S5.3). The opposite trend is observed for CA4 where the model preferred to predict those experiments to fail and then wrongly predict 18 experiments (see peak “b” in Figure S5.3). This can be explained by the fact that the dataset is not enriched enough in successful reactions using this coupling agent, in order words, the dataset is somehow unbalanced which has direct consequence on the model learning.

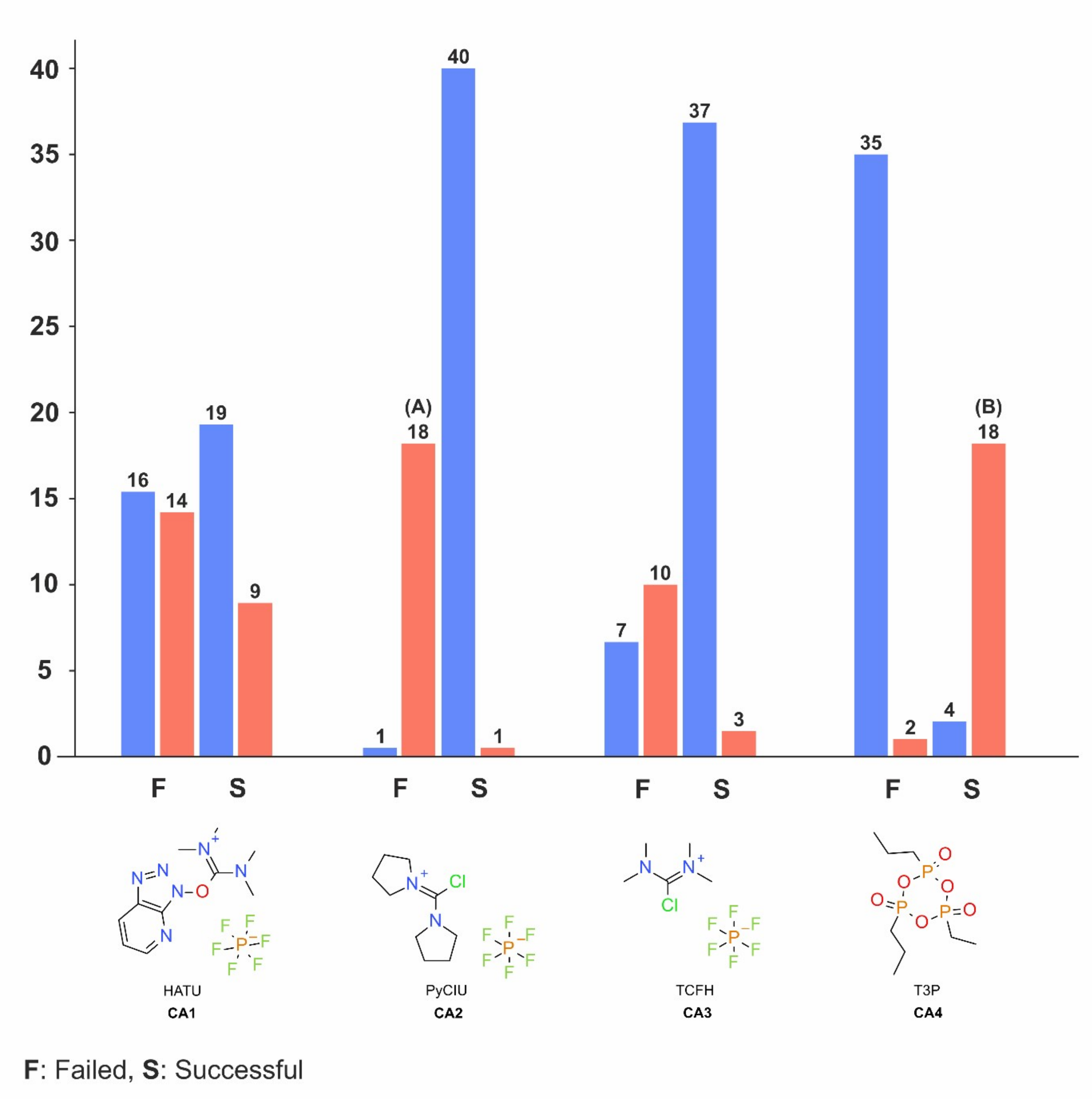

Figure S5.3. Accuracy of the prediction with respect to the coupling agent. The blue bars represent the number of experiments where the outcome (successful or failed reaction) have

been properly predicted by the model. Oppositely, the red bar represents the number of wrong predictions. The X-axis further informs if the well or badly predicted experiments belongs to successful (‘success’) or failed reactions (‘failed’).

S5.7 Percentage of conversion analysis

In the Figure S5.4 below, the average conversion percentage with respect to the products and two different predicted score thresholds, ‘Score >0.0’ (e.g. meaning no limitation using the score), ‘Score>0.8’ means that only the experiments having a score equal or greater than 0.80 are kept and would have been then considered experimentally. For clarity reason, the picture has been divided into Fig S5.4a and Fig S5.4b that represent 2 sets of 15 product correspond to 3 distinct acid each). Apart from some exceptions, it is clear that considering only higher scored experiments leads to an increase of the average conversion for most of the products. For some products, the average conversion largely increased when considering the higher ‘Score >0.80’ compared to no applying a threshold on the score. e.g. ‘Score > 0.00’. However, in two cases only, the products would have not been synthesized as none of the experiments received a ‘Score > 0.80’.

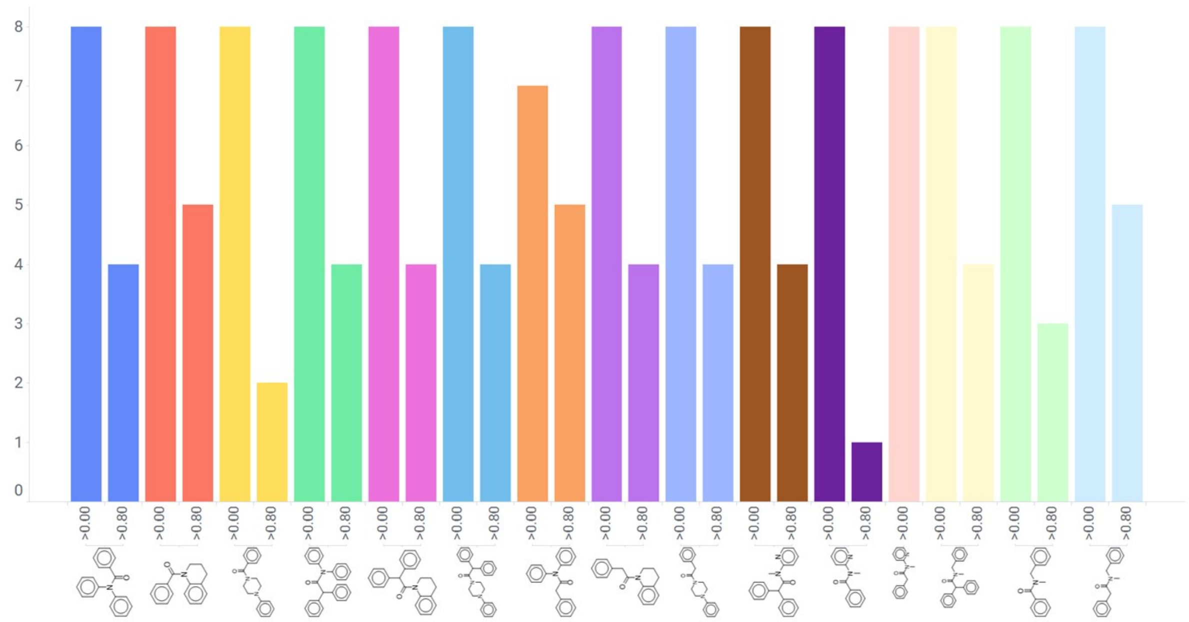

Figure S4.4a. Set 1: average conversion percentage with respect to the products at two different predicted score thresholds.

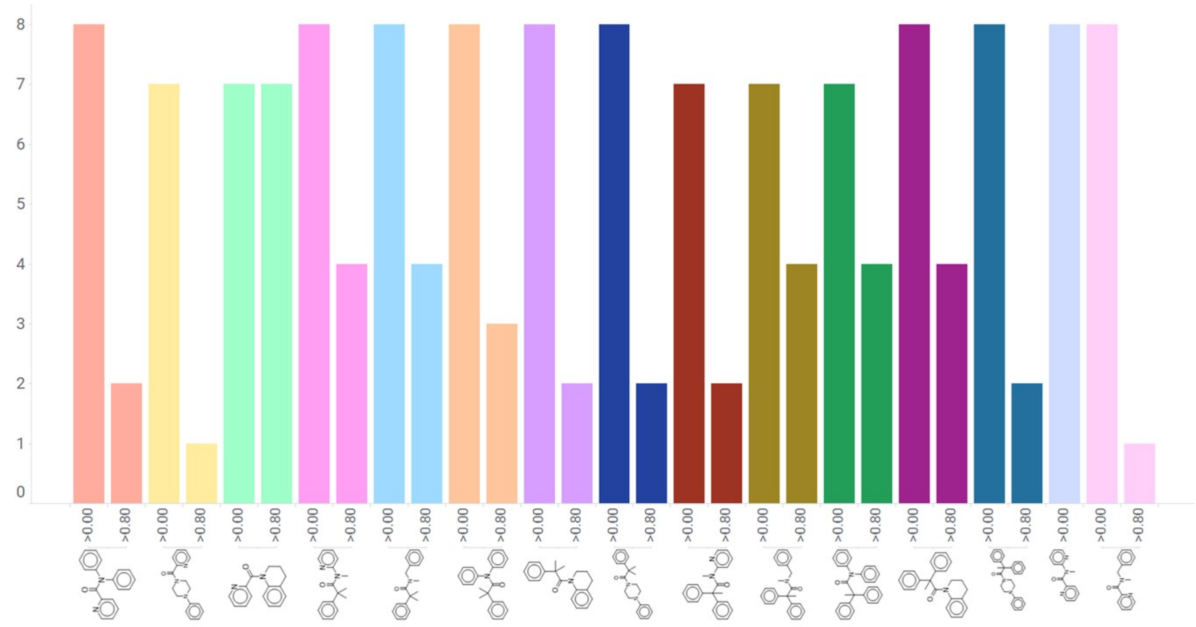

Figure S4.4b. Set 2: average conversion percentage with respect to the products at two different predicted score thresholds.

References

1. Wegner, J., S. Ceylan, and A. Kirschning, *Ten key issues in modern flow chemistry.* Chem Commun (Camb), 2011. **47**(16): p. 4583-92.
2. Fanelli, F., et al., *Contribution of microreactor technology and flow chemistry to the development of green and sustainable synthesis.* Beilstein J Org Chem, 2017. **13**: p. 520-542.
3. Gobert, S.R.L., et al., *Characterization of Milli- and Microflow Reactors: Mixing Efficiency and Residence Time Distribution.* Organic Process Research & Development, 2017. **21**(4): p. 531-542.
